# Biosynthesis of a clickable pyoverdine via in vivo enzyme engineering of an adenylation domain

**DOI:** 10.1186/s12934-024-02472-4

**Published:** 2024-07-24

**Authors:** Hélène Puja, Laurent Bianchetti, Johan Revol-Tissot, Nicolas Simon, Anastasiia Shatalova, Julian Nommé, Sarah Fritsch, Roland H. Stote, Gaëtan L. A. Mislin, Noëlle Potier, Annick Dejaegere, Coraline Rigouin

**Affiliations:** 1 CNRS, UMR7242 Biotechnologie et Signalisation Cellulaire, 300 Boulevard Sébastien Brant, 67412 Illkirch-Graffenstaden, France; 2 Université de Strasbourg, Institut de Recherche de l’Ecole de Biotechnologie de Strasbourg (IREBS), 300 Boulevard Sébastien Brant, 67412 Illkirch-Graffenstaden, France; 3 Département de Biologie structurale intégrative, Institut de Génétique et de Biologie Moléculaire et Cellulaire (IGBMC), Institut National de La Santé et de La Recherche Médicale (INSERM), U1258/Centre National de Recherche Scientifique (CNRS), UMR7104/Université de Strasbourg, Illkirch-Graffenstaden, France; 4 CNRS, UMR7140 Chimie de la Matière Complexe, Laboratoire de Spectrométrie de Masse des Interactions et des Systèmes, Université de Strasbourg, 4 Rue Blaise Pascal, 67082 Strasbourg, France

## Abstract

The engineering of non ribosomal peptide synthetases (NRPS) for new substrate specificity is a potent strategy to incorporate non-canonical amino acids into peptide sequences, thereby creating peptide diversity and broadening applications. The non-ribosomal peptide pyoverdine is the primary siderophore produced by *Pseudomonas aeruginosa* and holds biomedical promise in diagnosis, bio-imaging and antibiotic vectorization. We engineered the adenylation domain of PvdD, the terminal NRPS in pyoverdine biosynthesis, to accept a functionalized amino acid. Guided by molecular modeling, we rationally designed mutants of *P. aeruginosa* with mutations at two positions in the active site. A single amino acid change results in the successful incorporation of an azido-l-homoalanine leading to the synthesis of a new pyoverdine analog, functionalized with an azide function. We further demonstrated that copper free click chemistry is efficient on the functionalized pyoverdine and that the conjugated siderophore retains the iron chelation properties and its capacity to be recognized and transported by *P. aeruginosa*. The production of clickable pyoverdine holds substantial biotechnological significance, paving the way for numerous downstream applications.

**Supplementary Information:**

The online version contains supplementary material available at 10.1186/s12934-024-02472-4.

## Introduction

Non-ribosomal peptide synthetases (NRPS) are involved in the production of a large diversity of peptides that have high biotechnological and clinical values such as plant toxins (syringomycin), antibiotics (polymyxins, vancomycin), anticancer molecules (bleomycin) and siderophores [1, 2]. In order to produce new-to-nature active compounds and expand biotechnological applications, bioengineering of NRPS to reroute their machinery has attracted considerable attention [3]. NRPS are megaenzymes divided into modules working in an assembly-line fashion, where individual modules are responsible for the selection, activation and condensation of an adenylated amino acid into the growing peptide chain [4]. A canonical NRPS module is subdivided in three domains, namely (i) the adenylation (A) domain, responsible for the selection and activation of the amino acid substrate, (ii) the thiolation (T) or peptidyl-carrier protein domain bearing a 4ʹ-phosphopantetheine group that covalently attaches to the growing peptide chain, and (iii) the condensation (C) domain responsible for peptide bond formation between the activated residue and the growing non-ribosomal peptide (NRP). The last module of the assembly-line is generally equipped with an additional thioesterase domain to catalyze the removal of the peptide from the assembly line, either via hydrolysis or cyclisation [5]. Various strategies are employed to create diversity and generate new peptides from these assembly lines. Precursor-directed biosynthesis relies on the naturally relaxed specificity of some enzymes, whereas enzyme engineering aims at either modifying the substrate specificity of the enzyme to accept new substrates or at performing domain and module swapping to create new peptides [6, 7].

The adenylation domain (A-domain) plays a central role in substrate recognition and activation. It is responsible for selecting the appropriate amino acid to be incorporated by the module [8] through two different reactions: the adenylation of the substrate with adenosine-5ʹ-triphosphate (ATP), followed by the transfer and fixation of the amino-acyl residue on the T-domain. The adenylation is secured by a conserved catalytic Lys residue that ensures covalent bond formation between the amino-acid and the ATP group [9]. The substrate specificity of the domain is ensured by a “specificity-conferring code” comprised of eight residues located in the binding pocket, first identified by sequence alignments [10, 11] and further reinforced by structural data [12]. The modification of these residues can change substrate specificity making this approach relevant for the production of peptides analogs [8, 13–15]. Although the A-domain displays a specificity code to incorporate a specific substrate, some A-domains are more permissive and can incorporate several substrates [16]. Most of the characterized A-domains are specific for proteinogenic amino-acids, although there have been reports of A-domains naturally incorporating non-proteinogenic amino-acids [17] or β-keto acids [18]. A category of non-canonical substrates that can be valuable for incorporation into a peptide is azide-containing amino acids, a chemical group with high biotechnological value since it can be used for bioorthogonal click-chemistry reactions. The most widely used click reactions are copper-catalyzed azide-alkyne cycloadditions (CuAAC) [19, 20], and strain-promoted azide-alkyne cycloaddition (SPAAC) [21]. Success has been achieved in incorporating azide-functionalized amino acids into NRP using precursor-directed biosynthesis strategies. This strategy takes advantage of the natural promiscuity of some A-domains and has led to the in vivo production of modified peptides [22, 23]. However, for A-domains displaying lower substrate promiscuity, mutagenesis is required to modify their substrate specificity. While positive outcomes have been attained in vitro, it is rarely combined with successful production in vivo of the corresponding clickable peptides [14, 24, 25].

**Fig. 1:**
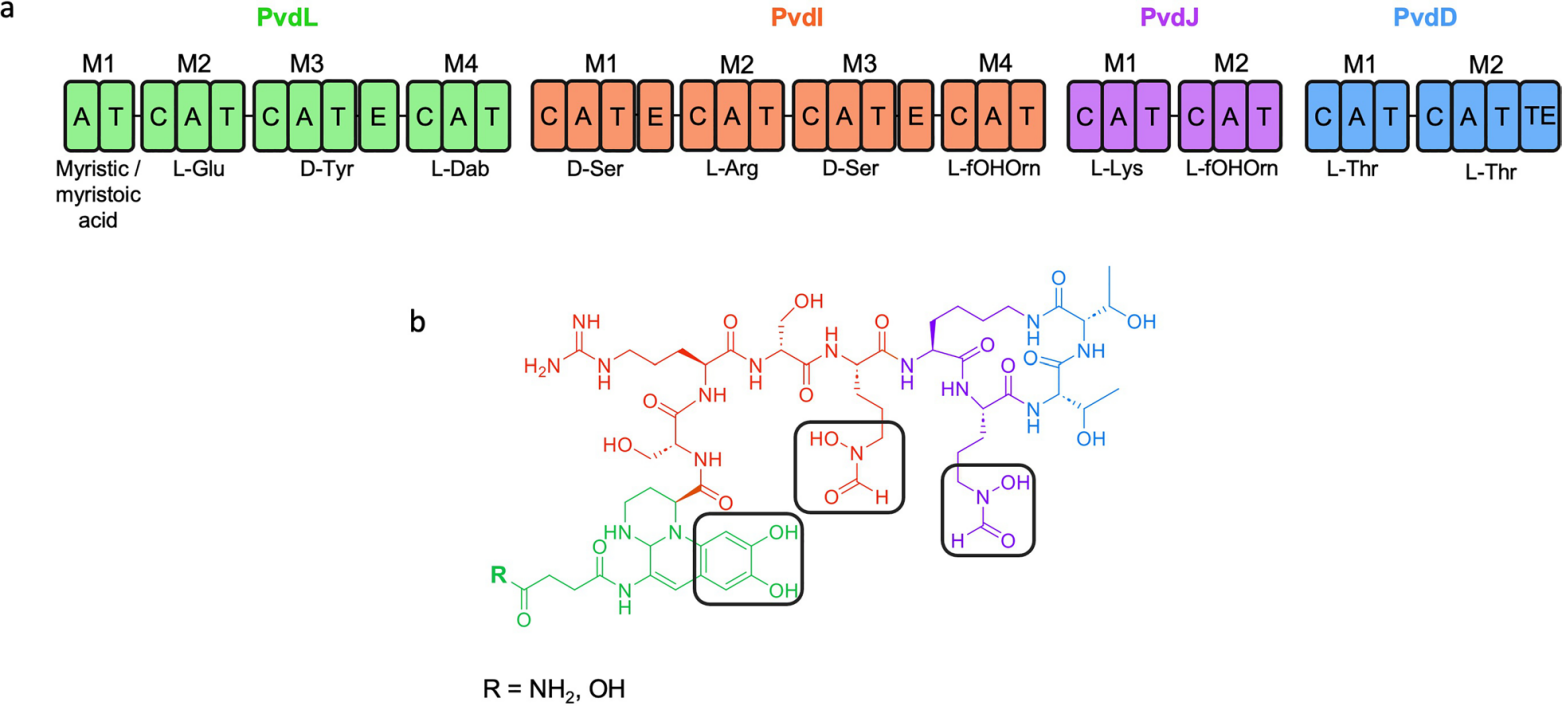
**a** NRPS involved in PVD biosynthesis C: Condensation domain; A: Adenylation domain; T: Thiolation domain; E: Epimerisation domain; TE: Thioesterase domain. l-Dab: l-2,4-diaminobutyrate; l-fOHOrn: l-N5-formyl-N5-hydroxy-ornithine (**b**) Structure of the mature PVD. The colors represent the relative contribution of each NRPS during synthesis: PvdL (green), PvdI (orange), PvdJ (purple) and PvdD (blue). The chelating groups are highlighted in black squares. R shows the two most frequently observed pyoverdines produced by *P. aeruginosa* PAO1, with the lateral chain being succinamide (NH2) or succinic acid (OH)

Pyoverdine (PVD) is an NRP belonging to the class of siderophores and produced by fluorescent *Pseudomonas.* PVD is secreted by the cell in the extracellular medium where it chelates iron with very high affinity (10^32^ M^−1^) [26]. Since iron is essential for sustaining life, siderophores represent the primary strategy for bacteria to access iron. PVD is synthesized in the cytoplasm by four NRPS, PvdL, PvdI, PvdJ and PvdD (Fig. 1a) and matured in the periplasm [27]. The final PVD peptide consists of a dihydroxyquinoline-type chromophore, a peptide chain of variable length and conformation, and a side chain composed of a succinic acid or its monoamide derivative (Fig. 1b). The uptake of iron-loaded pyoverdine is achieved by dedicated and specific outer-membrane transporters [27]. In addition to playing an important role for survival, proliferation and virulence of the bacteria, PVD could be exploited in biotechnology in various fields [28]. In medicine, it has encouraging prospect in bio-imaging, biosensing, and diagnosis [29, 30]. The ability of PVD to cross bacterial membranes using specific transporters has led to the synthesis of siderophore-antibiotic conjugates that are able to hijack the iron-acquisition pathway to deliver antibiotics inside the cell [31, 32]. PVD could also be suitable for agricultural purposes, supporting plant growth or fighting plant pathogens [33, 34] and has great potential in soil bioremediation for bioleaching [35] or bioweathering [34, 36]. Creating analogs of PVD has the capacity to broaden its structural diversity and introduce novel properties associated with new applications [7]. Only one total synthesis of PVD has been reported so far [37]. This synthesis highlighted that chemical synthesis of complex metabolites suffers from the use of toxic solvents, requires numerous successive steps and displays a very low overall yield. Enzyme engineering by domain swapping has been applied to PvdD to generate PVD analogs [38]. While their study establishes a proof of concept that PvdD is a good target to create PVD diversity, the generated analogs only harbored proteinogenic amino acids. PvdD incorporates the last two amino acids (Thr) of PVD. This part of the peptide is neither involved in the formation of the chromophore, in iron chelation, nor in the recognition by the outer membrane transporters that import the ferri-PVD complex [39]. Thus, it appears to be a good target for modification without impacting PVD function. In this study, we performed site-directed mutagenesis, guided by molecular modeling, on the first A-domain of PvdD (PvdD(A1)) to modify the substrate specificity toward an azide-functionalized amino acid. We demonstrate the successful engineering of PVD pathway allowing for the first time the biosynthesis of azide-functionalized PVD.

## Results

### Exploration of PvdD(A1) sequence and structure: identification of two residues to target for a change in substrate specificity

**Fig. 2:**
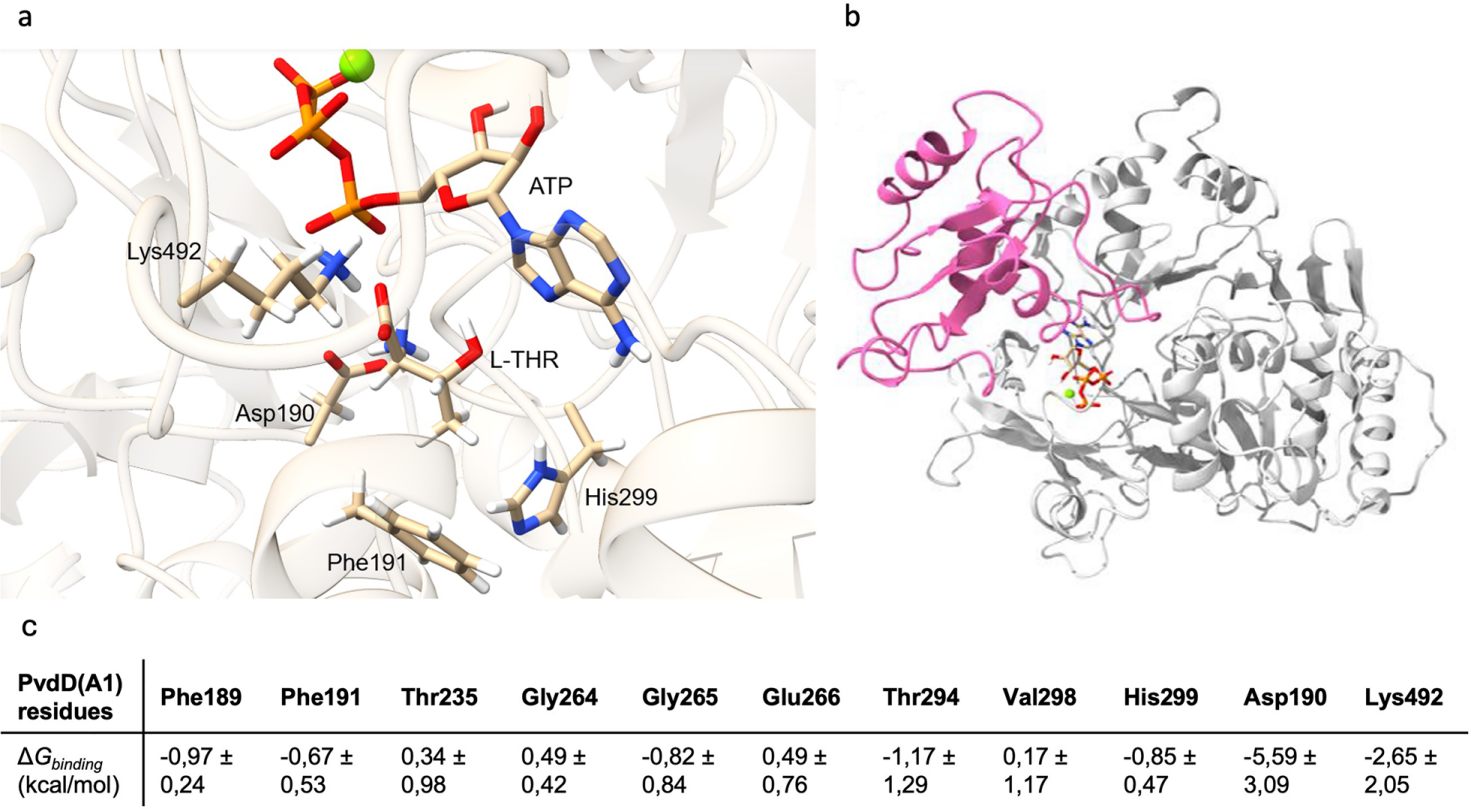
3D representation of PvdD(A1). **a** 3D model of the substrate binding pocket of PvdD(A1) interacting with ATP (orange), a magnesium ion (green) and the natural substrate l-Thr (l-THR). The conserved Asp190 and the catalytic Lys492 are shown, as well as the two key residues Phe191 and His299. **b** PvdD(A1) with the Acore subdomain (white) and Asub subdomain (pink). **c** MM/PBSA calculations of free binding energy (kcal/mol) of the residues of PvdD(A1) for the stabilization of l-Thr

PvdD(A1) active site comprises the catalytic residue Lys492, the conserved Asp190 involved in the adenylation activity and the variable residues stabilizing the side chain of the amino acid substrate. These variable residues involved in the substrate recognition of l-threonine were first identified through sequence alignments. We worked under the hypothesis that a highly conserved residue in threonine specific A-domains, that is very rarely found in non-threonine A-domains, is a key residue for l-Thr stabilization. We focused in priority on the eight specificity-conferring positions (Additional file 1: Fig. S1). This sequence analysis identified 2 positions, Phe191 and His299, as potentially interesting for modification. To give further basis to this choice, we constructed a homology-based structural model of PvdD(A1). In the model, the l-Thr substrate, the ATP and the Mg^2+^ ion were placed in the cavity (Fig. 2a) by comparative docking, using the homologous *Streptomyces* SP.OH-5093 A-domain structure with a bound l-Thr (PDB-ID:5N9X) [40]. As described in the literature, the α-amino group and α-carboxyl groups of the l-Thr substrate are located at the entrance of the pocket, recognized respectively by Asp190 and the catalytic residue Lys492. The 8 residues responsible for the substrate specificity [11] are located in the stable Acore subdomain, and the Lys492 is located on the dynamic Asub subdomain (Fig. 2b). The Asub domain undergoes a conformational change after substrate selection to shift from an open conformation to an active adenylation conformation [41]. Our model showed that the aromatic residues Phe191 and His299, located on the sides of the pocket, promote the stabilization of the l-Thr short side chain. Molecular mechanics Poisson-Boltzmann surface area (MM/PBSA) calculations of binding free energy allowed us to determine the individual contribution of the residues located in the A1-domain towards stabilization of the l-Thr substrate in the pocket (Fig. 2c). The free-energy analysis clearly shows the determinant role of Asp190 and Lys492 in stabilizing the substrate, and indicate five residues that participate in stabilizing l-Thr, including Phe191 and His299. This strengthens the sequence analysis and expands on previous analysis of PvdD(A)1 specificity [42].

### Single amino acid change in PvdD(A1) lead to the production of PVD analogs

**Fig. 3:**
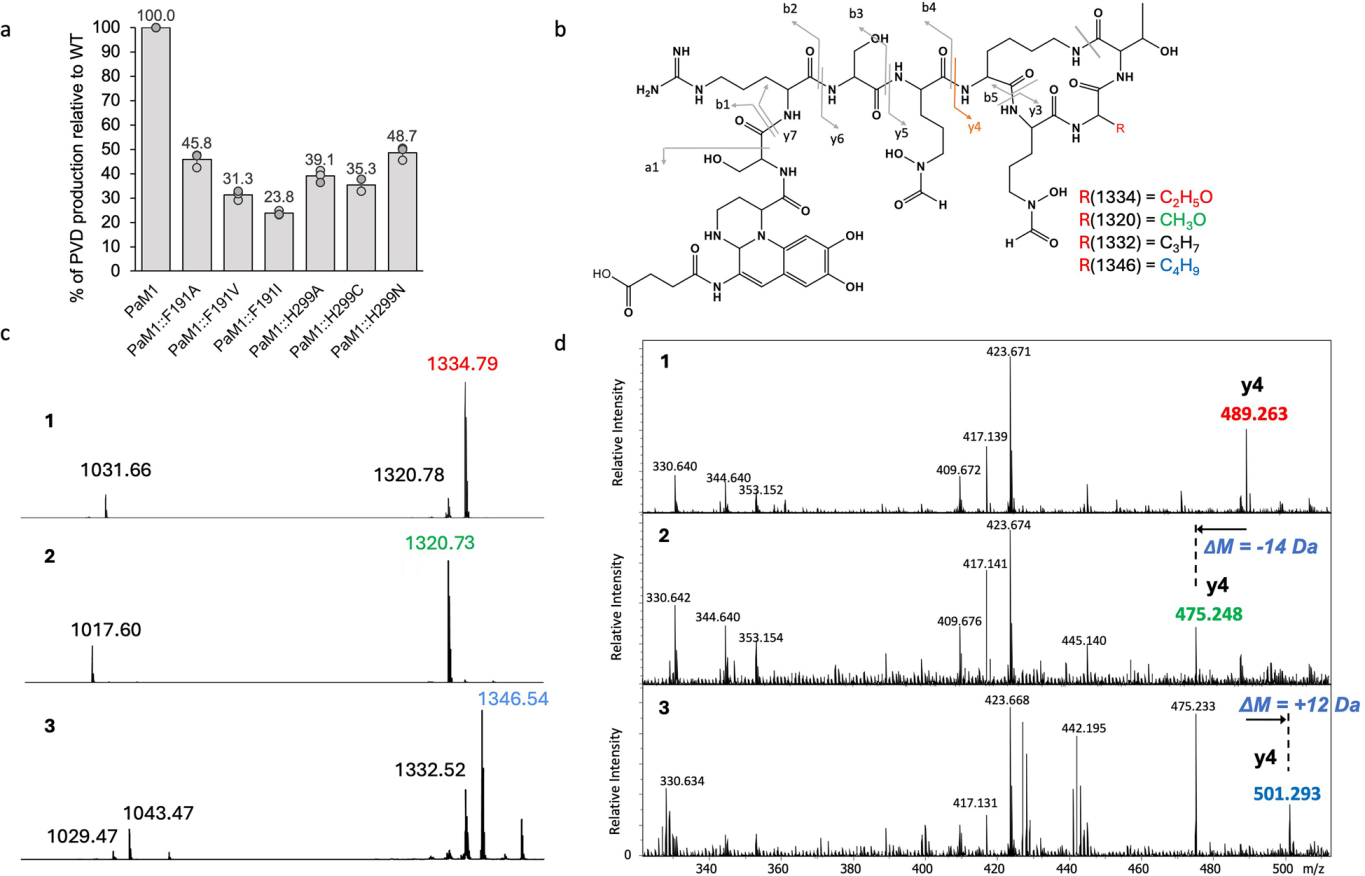
PVD production by mutant strains in CAA. **a** % of PVD produced by the mutants after 48 h of culture compared to PaM1 **b** ESI–MS/MS ions identification on PVD structure. The red « R» shows the different side chains of the amino acid incorporated by PvdD mutants. **c** MALDI mass spectra of PVD produced by PaM1 (1), the mutants Phe191Val (2) and His299Ala (3). **d** ESI–MS/MS spectrum of the three corresponding PVD major species detected in **c**. Doubly-charged ions at *m/z* 667.8 (1), 660.8 (2), and 673.8 (3) have been respectively selected and submitted to MS/MS experiments at 30 eV collision energy. A zoom in the *m/z* 320–510 range is shown here for better visualization of the discriminant fragment ions between the PaM1 and the mutants (highlighted in red for PVD-Thr, green for PVD-Ser or blue for PVD-Leu/Ile). The mass shift observed for the y4 ions is indicated by a black arrow and correspond to the mass shift observed for the main peak by MALDI-MS

We built a set of mutants at position Phe191 and His299 from the parent strain *P. aeruginosa* PaM1 [36], and directly monitored the impact of the mutations on the PVD produced. Three mutants were constructed at the Phe191 position (Phe191Ala, Val, Ile) and three mutants at the His299 position (His299Ala, Val, Cys). The mutations had no effect on bacterial growth (Additional file 1: Fig. S2), however a decrease in PVD production was observed for all mutants (Fig. 3a). The PVD produced by the strains were purified and analyzed by MALDI mass spectrometry. For the PaM1 strain, the native PVD was detected at the expected *m/z* 1334.79 (Fig. 3c-1). A compound resulting from a retro-Diels Alder reaction of the PVD chromophore was also observed (*m/z* 1031.66, Fig. 3c-1) [43]. Surprisingly, a second PVD with a lower mass (*m/z* 1320.78, Fig. 3c-1) was also detected, albeit at a lower intensity than the native one. Additional tandem mass spectrometry (MS/MS) experiments were conducted by electrospray ionization (ESI) [44] to further characterize these species. Only the y4 (*m/z* 489.263) and y7 (*m/z* 890.465) ions, corresponding to the C-terminal part of PVD (Fig. 3d-2 and Additional file 1: Table S1), were found displaced at − 14 amu showing that this unexpected PVD corresponds to a variant of the native product where l-Ser has been incorporated instead of l-Thr. This relaxed specificity of the native NRPS was not expected, as previous in vitro experiments [42] did not indicate substantial incorporation of l-Ser. It is however coherent with the bioinformatics and energetic analysis of our modelled structures, that l-Ser binds to PvdD(A1), albeit with less favorable interactions than l-Thr (data not shown).

The MALDI-mass spectrum of the PVD produced by the mutant Phe191Val differed from that of PaM1 with one main peak observed at *m/z* 1320.73 (Fig. 3c-2). The ESI MS/MS analysis displayed an identical fragmentation pattern of the corresponding doubly-charged ion at *m/z* 660.8 produced by the mutant Phe191Val and by PaM1 (Additional file 1: Fig. S4 and Table S1) showing that the mutant Phe191Val produced the same PVD-Ser as the wild-type strain. To quantify the amount of PVD-Ser produced by the mutant with respect to the WT, we performed a titration experiment by MALDI-MS (Additional file 1: Fig. S5) which showed that the Phe191Val mutant strain produces a small amount of PVD-Ser, like the wild-type PaM1, but is unable to produce PVD-Thr. Interestingly, the mutant Phe191Ala showed the same PVD production profile as the WT parent strain PaM1, while the mutant Phe191Ile showed the same profile as the mutant Phe191Val (Additional file 1: Fig. S6).

The MALDI mass spectrum of the His299Ala mutant displayed a heterogeneous signal with two mains peaks, at *m/z* 1332.52 and 1346.54 (Fig. 3c-3). ESI–MS/MS experiments of these two peaks (Fig. 3d-3 and Additional file 1: Fig. S3, Table S1) showed again that the mass modifications took place in the C-terminal part of the peptide, confirming that the His299Ala mutation led to the incorporation of other proteinogenic amino acids, i.e. l-Val (leading to PVD-Val, *m/z* 1332.52 Fig. 3d-3) and l-Leu or l-Ile (leading to PVD-Leu/Ile, *m/z* 1346.54 Fig. 3d-3). As observed before for the mutants at position Phe191, some mutants at position His299 displayed PaM1 like PVD production (His299Asn) while some incorporated new amino acids (mutant His299Cys, see Additional file 1: Fig. S6).

### The His299Ala mutant strain produces an azide-functionalized PVD

**Fig. 4:**
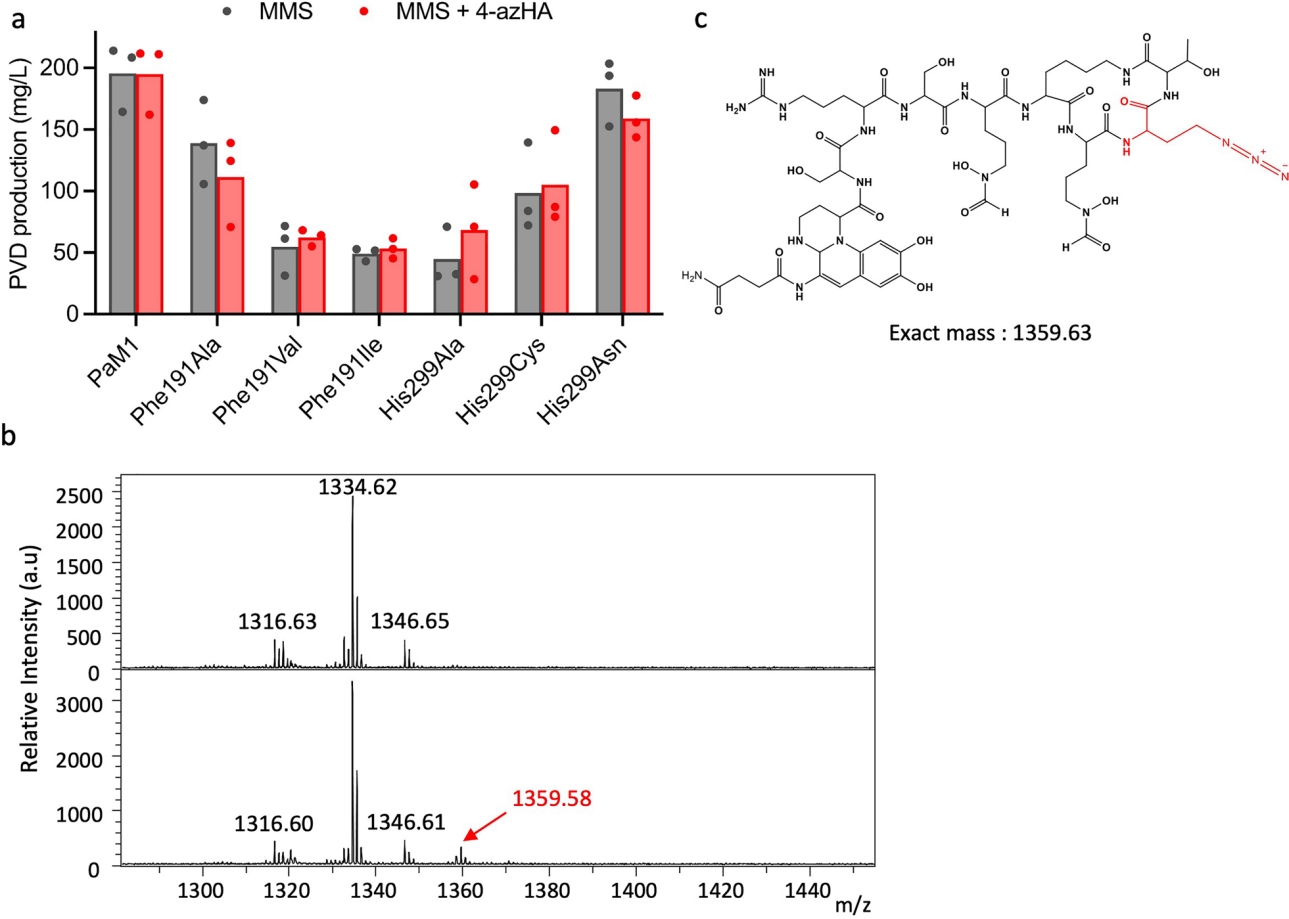
Incorporation of 4-azHA in the biosynthesis of PVD. **a** PVD production by the PaM1-derived mutant strains in MMS (grey) and MMS supplemented with 4-azHA (red). **b** MALDI mass spectrum of the PVD produced by the mutant His299Ala cultivated with (lower frame) or without (upper frame) 4-azHA supplementation. A signal at m/z 1359.58 whose molecular mass corresponds to the expected azide-functionalized PVD (PVD-azHA) is highlighted in red. **c** Structure of PVD-azHA after incorporation of 4-azHA into PVD biosynthesis

The capacity of the Phe191 and His299 mutants to incorporate an azide-functionalized amino acid was tested in a minimal medium with succinate as the carbon source (MMS) by supplementing the medium with 4-azido-l-homoalanine (4-azHA) as sole supply of exogenous amino acid. The PVD profile of PaM1 and Phe191 mutants were identical in MMS to a production in CAA medium (Fig. S7). The His299Ala and His299Cys mutants were found to mainly produce PVD-Thr and PVD-Leu/Ile in MMS and no PVD-Val (Fig. 4b). The presence of 4-azHA reduced the growth rate of all tested strains, indicating a slight toxic effect of the azide-functionalized amino acid at the chosen concentration (Additional file 1: Fig. S2-b). However, for all the mutants, the addition of 4-azHA to the culture does not affect the global yield of PVD (Fig. 4a), indicating that this growth rate reduction is not a major issue.

The incorporation of 4-azHA by the mutants was monitored by analysis of the produced PVDs by MALDI-MS. For the three mutants at position Phe191 and two of the mutants at position His299 (His299Cys and Asn), no difference in the MALDI spectrum of the PVD produced was seen when grown with or without 4-azHA, suggesting that these mutants are not able to incorporate 4-azHA into PVD. Remarkably, for the His299Ala mutant, a new peak at m/z 1359.58 was observed, corresponding to the expected mass of azide-functionalized PVD (PVD-azHA) (Fig. 4b and c). No additional MALDI signal is observed if the parent strain PaM1 is grown in minimal medium supplemented with 4-azHA, nor with the His299Ala mutant cultivated in minimal medium in absence of 4-AzHA (Fig. 4b).

We then built the 3-D model of the His299Ala A1-domain and performed molecular dynamics simulations of both wild type (WT) and His299Ala A1-domains in a complex with 4-azHA. The WT A1-domain docked with 4-azHA showed that the residue His299 at the bottom of the pocket could interfere with the azide moiety through a steric interaction due to the rigidity of the azide group and the large volume of the aromatic moiety of His. (Additional file 1: Fig. S8a). These results were associated with an unfavorable contribution of the residue His299 to the binding free energy as indicated by MM/PBSA calculations (Additional file 1: Fig. S8c). In the His299Ala mutant, the replacement of His299 by Ala removed the steric hindrance, allowing the docking of 4-azHA at the bottom of the pocket (Additional file 1: Fig. S8b). The main chain of the amino acid is correctly positioned at the entrance of the pocket, allowing the interaction of the α-carboxyl with the catalytic Lys492 residue. Additional convergent data for 4-azHA binding in the WT or His299Ala substrate binding pockets by using MM/PBSA is provided in Additional file 1: Fig. S8c. The pocket of the mutant His299Ala presented a significantly lower global binding free energy for 4-azHA compared to WT (-54%), indicating a better stabilization of 4-azHA in our model.

Another important factor for an effective adenylation activity is the flexibility of the Asub subdomain. This flexibility ensures the transition from the “open” state to the “adenylation” conformation. During this transition, the Asub subdomain, which contains Lys492, rotates to align itself with the substrate securely positioned in the Acore subdomain, thereby enabling catalytic activity [41]. To verify the flexibility of the A-domain when complexed with 4-azHA, we calculated the root-mean-square fluctuations (RMSF) from the molecular dynamics simulations (Additional file 1: Fig. S8d, e). We found that the His299Ala mutation raised the global flexibility of the Asub subdomain.

### Effective click-chemistry of the PVD-azHA for the production of a PVD-oxazolidinone conjugate

**Fig. 5:**
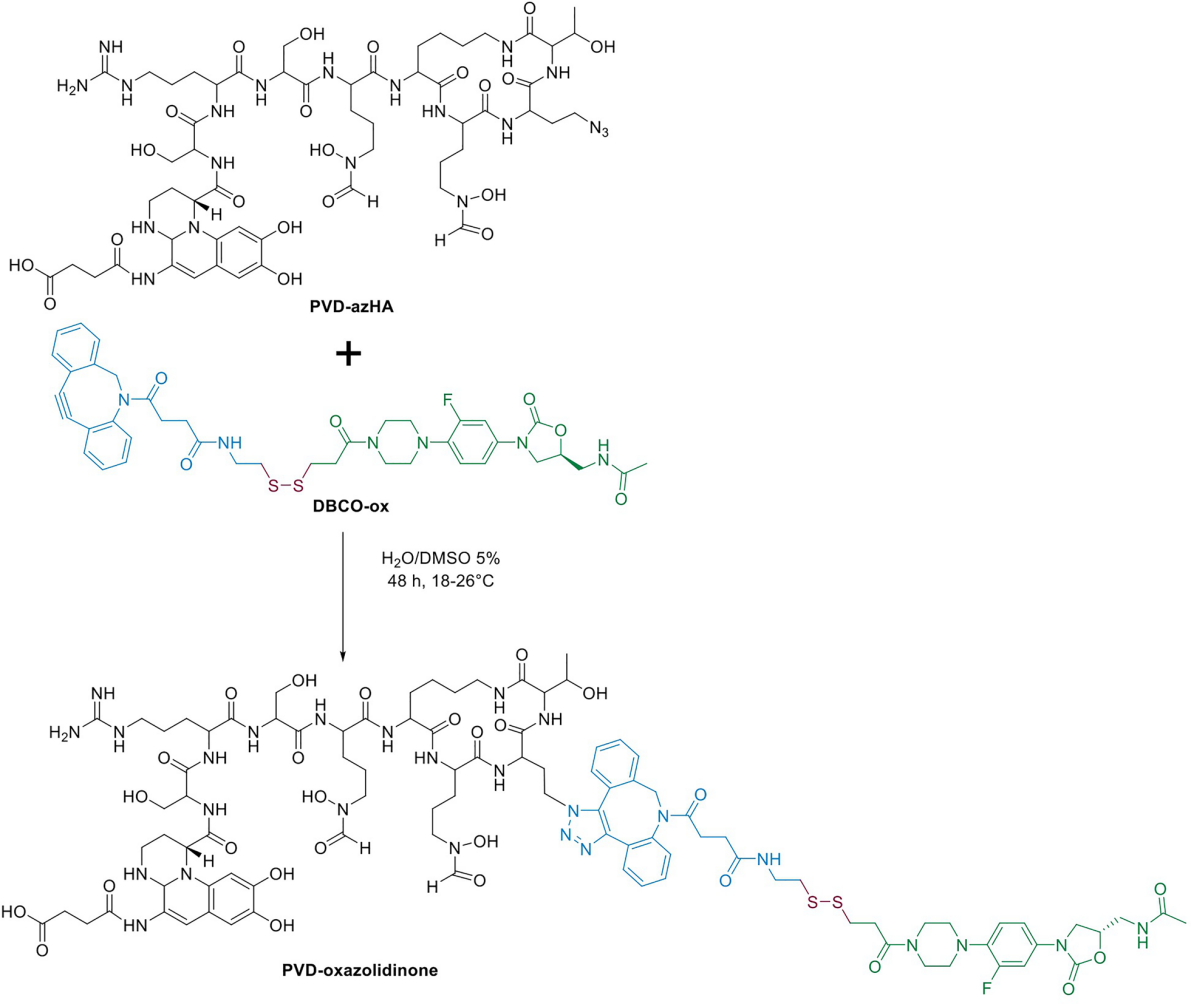
SPAAC to synthesize the PVD-oxazolidinone conjugate. In blue is represented the DBCO-linker, in purple the disulfide bond and in green the oxazolidinone antibiotic

**Fig. 6:**
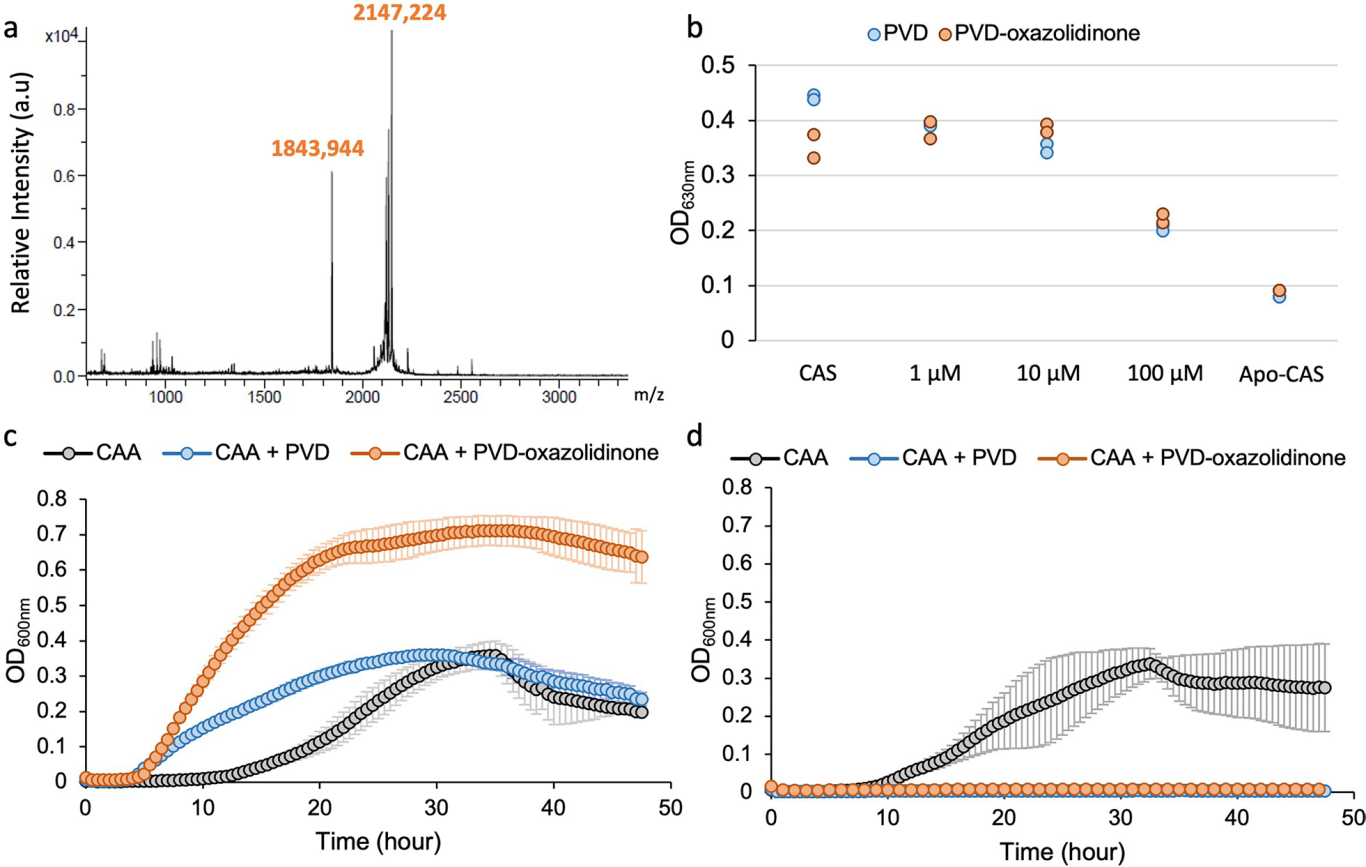
Characterization of the PVD-oxazolidinone conjugate. **a** MALDI-MS spectrum of the purified PVD-oxazolidinone conjugate. **b** CAS-assay showing the ability of PVD-Thr (blue) and PVD-oxazolidinone (orange) to chelate iron. Growth assay in minimal medium CAA (black) of the strain PAO1Δ*pvdF*Δ*pchA* (**c**) and strain PAO1Δ*pvdF*Δ*pchA*Δ*fpvA*Δ*fpvB* (**d**) in the presence of PVD-Thr (blue) or PVD-oxazolidinone (orange)

To establish the proof of concept that PVD-azHA can be efficiently conjugated to an alkyne-functionalized molecule, we choose to perform a click reaction using an antibiotic functionalized with a dibenzocyclooctyne (DBCO). Trojan horse strategies using siderophores as cargo have been shown to broaden the spectrum of antibiotics [45, 46] which for oxazolidinones antibiotics is usually limited to Gram-positive. Previous studies have provided evidence that, when conjugated to a siderophore, the oxazolidinone antibiotic eperezolid becomes active against Gram-negative bacteria [47]. The release of the oxazolidinone into the periplasm was shown to be crucial for the activity in Gram-negative bacteria [48, 49]. Consequently, the periplasmic fate of PVD in *P. aeruginosa* [50] provides an opportunity to vectorize oxazolidinones into this critical bacterium. For this purpose, the PVD-azHA was connected to an oxazolidinone through a linker containing the potentially in vivo cleavable disulfide bond, and functionalized with a DBCO (DBCO-ox**,** synthesis described in Additional files 1: Fig. S16 and 2). The conjugation between PVD-azHA and the DBCO-ox followed a SPAAC reaction (Fig. 5). The conjugate PVD-oxazolidinone (PVD-ox) was detected by MALDI-MS, as expected, at *m/z* 2146.45, as well as the corresponding Retro-Diels Alder rearrangement at *m/z* 1843.26 (Additional file 1: Fig. S9). It was then successfully separated from other PVD species (Fig. 6a) by size exclusion chromatography. The amount of PVD-ox obtained was estimated to be 5 mg from a 1L culture of the His299Ala mutant.

The PVD-ox was tested for its antibacterial activity through a minimal inhibitory concentration assay (MIC), but we measured a MIC value > 512 µM for PVD-ox and for DBCO-ox against *P. aeruginosa* strain PAO1, which suggests that the PVD-ox is not active against *P. aeruginosa*. To verify that the PVD-ox can still chelate iron and be recognized by PVD-specific transporters, we performed a Chrome-azurol S colorimetric assay using PVD as a control. Our results showed that PVD-ox chelates iron with a profile similar to that of PVD (Fig. 6b). In addition, we monitored the capacity of PVD-ox to be transported by the PVD-specific uptake pathway of *P. aeruginosa*. To do so, we used the strain PAO1Δ*pvdF*Δ*pchA*, a PAO1-derived strain unable to produce its own siderophores PVD and pyochelin, and we evaluated its ability to grow in minimal medium supplemented or not with PVD or PVD-ox (Fig. 6c). The addition of either PVD or PVD-ox resulted in growth promotion of the strain, suggesting that PAO1Δ*pvdF*Δ*pchA* uses PVD-ox as a siderophore. The highest growth stimulation observed with PVD-ox can be explained by the presence of iron chelated by this siderophore, acquired during the multiple purification steps. To verify the transport of the conjugate, we constructed the strain PAO1Δ*pvdF*Δ*pchA*Δ*fpvA*Δ*fpvB* lacking both PVD transporters, FpvA and FpvB and followed its growth in the presence of PVD or PVD-ox (Fig. 6d). We found a complete growth inhibition in the presence of both siderophores proving that the bacteria cannot access iron anymore. These results prove that PVD-ox, similarly to PVD, chelates iron in the minimal medium and is transported into the bacteria through the PVD-specific uptake pathway. This discovery opens up new perspectives for conjugating PVD with various antibiotics for their delivery to the critical pathogen *P. aeruginosa.*

## Discussion

We have described in this study the successful engineering of an adenylation domain for in vivo incorporation of a clickable amino acid into an NRP. We applied the strategy to the biosynthesis of the siderophore PVD in order to facilitate its conjugation and expand its applications. Our engineering efforts focused on PvdD, one of the four NRPS enzymes involved in PVD biosynthesis. Calcott *et al.* previously described a successful domain swapping strategy applied to PvdD A-domain [38, 51] and, while preparing this manuscript, an elegant strategy involving metagenomics substitution of NRPS domains that led to the production of PVD analogs appeared [52]. However, the generated analogs only contain proteinogenic amino acids that are not conjugatable, limiting their downstream applications. In our study, we managed to produce diverse PVD analogs with proteinogenic amino acids and for the first time with a non-canonical amino acid. We discovered that *P. aeruginosa* PaM1, naturally produces two types of PVD and we provided evidence that the peptide sequence variation occurs at the C-terminal end of the peptide as a result of tolerant substrate specificity of PvdD for both l-Thr and l-Ser. The adenylation activities of several A-domains incorporating l-Thr residue have been tested in vitro, yielding contrasting results. DhbF2, a NRPS involved in bacillibactin synthesis, was described to be highly specific to l-Thr [53], whereas purified MbtB, a NRPS involved in mycobactin synthesis, was able to activate l-Ser in addition to l-Thr [54]. In an early work of 2003 [42], Ackerley and colleagues did not measure in vitro a significant activity of PvdD(A1) toward l-Ser, which is discordant with our findings and could be explained by the difference of the methods used to monitor PvdD activity.

Several studies have reported the in vitro engineering of A-domain to change substrate specificity and most of them have used engineering strategies supported by molecular modeling [8, 14, 55]. A successful example is the study of Kries* et al*. [14] that showed that a single amino acid change in the l-Phe activating A-domain of GrsA, the tridomain initiation NRPS involved in gramicidin S biosynthesis, led to the adenylation of non-natural amino acids functionalized with azide and alkyne group. The equivalent mutation introduced into similar NRPS, either in the initiation TycA or an elongation module of TycB also led to the incorporation of l-Phe analogs with alkyne, halogen and benzoyl substituents into a reconstituted in vitro biosynthesis of the corresponding tyrocidine A peptide [25]. By sequence and structure alignment of the GrsA and TycA A-domains with PvdD(A1), we identified that the residue they mutated to modify the substrate specificity corresponds to the residue Trp194 in PVD(A1). This is one of the eight positions of the “specificity code” and one that is poorly represented in A-domains not specific for Thr but frequently present in Thr specific A-domain (Additional file 1: Fig. S1). However, in our system, the mutation of Trp194Ser or Lys did not lead to the incorporation of clickable amino acids into PVD structure. This indicates that substrate specificity is controlled by several amino acids in the pocket, and that modification strategies effective in one A-domain do not always translate in homologous cases in a straightforward manner. These results provide evidence that a combination of mutations at several of the eight positions of the specificity code is necessary to guide the specificity of an A-domain toward a particular amino acid [56, 57]. Consequently, this work opens up new opportunities for further engineering of PvdD(A1) to improve its specificity for 4-azHA and enhance PVD-azHA production.

Moreover, we opted to work in vivo by directly introducing the mutation into the bacterial genome and monitoring the resultant PVD structure. In vitro assay monitoring of PvdD A-domain activity could bring better insight on the catalytic properties of the mutated A-domain [9, 58]. Nonetheless, relying on in vitro results from single A-domain assays to engineer an in vivo assembly line that can generate NRP analogs is challenging because protein–protein interactions in NRPS machinery are essential for substrate processing [41]. For this reason, very few examples describe the successful in vivo incorporation of non-canonical amino acids by engineered NRPS [17, 56]. Conducting high-throughput assays in the context of in vivo engineering poses challenges and usually results in the exploration of small-sized mutant libraries. Nevertheless, this approach gains greater relevance when using cells as factories for the production molecules of interest [59].

Finally, we used the PVD-azHA produced by the mutant strain to synthesize a PVD-antibiotic conjugate and demonstrated that, even if no antimicrobial activity is measured, the PVD-ox chelates iron(III) and is efficiently transported into the bacterium. This opens the possibility to test several classes of antibiotics and to investigate the fate of the antibiotic once in the periplasm. The inefficiency of our compound against *P. aeruginosa* can be explained by the possibility that the reduction of the disulfide bound did not occur even if has been shown to be cleaved in other critical Gram-negative pathogens [60]. Alternative strategies using antibiotics with periplasmic targets could be implemented [32, 61], as well as the use of alternative linkers to release the antibiotic in the periplasm such as hydrolysable or labile spacer arms [47, 62].

Overall, our findings demonstrate the in vivo functionalization of PVD, paving the way for its conjugation to various types of molecules. This is particularly significant since we achieved a yield of approximately 5 mg of conjugate from 1 L of bacterial culture, prompting consideration for optimization through bacterial and bioprocess engineering. This breakthrough will expand the range of properties associated with this chelator. Our study provides compelling evidence that employing A-domain engineering guided by a molecular modeling approach is a potent strategy for in vivo incorporation of clickable amino acids in NRPs.

## Experimental section

### Construction of the *P. aeruginosa* mutant strains

All mutants were constructed in the strain *P. aeruginosa* PaM1, a PAO1-engineered strain modified to produce PVD through arabinose induction [36]. PaM1 has the exact same PVD production profile as the WT strain PAO1 (unpublished data). For these mutants, a genetic region of 1883 bp containing the A1 domain of *pvdD* from PAO1 was cloned into the pEXG2 plasmid [63] using NEBuilder® HiFi DNA Assembly (New England Biolabs, USA), following the manufacturer's instructions. Briefly, the plasmid was linearized and the genetic region of PvdD(A1) was amplified by PCR, using primers harboring overlapping regions recognizing each other (Additional file 1: Table S2). *Escherichia coli* Top10 competent strains were transformed with the assembly and the obtained plasmid pEXG2::*pvdD*(A1) was sequenced by Sanger sequencing for verification. To insert the desired mutations, the pEXG2::*pvdD*(A1) plasmid was PCR-amplified using error-inducing primers (Table S2). The residue Phe191 was replaced by other hydrophobic amino acids with lower steric hindrance with the aim to generate more space in the substrate binding pocket. For the residue His299, a larger panel of amino acid characteristic was tried to try to reduce steric hindrance (with Ala) and alter polarity and charge (with Cys and Asn). TOP10 cells were then transformed with this plasmid and used for triparental conjugation with *P. aeruginosa* PaM1 as the receiver strain and *E. Coli* HB101 pK2013 as the helper strain [64]. A two-step process allowed allelic exchange between the vector and the chromosome mediated by homologous recombination [65]. The mutated clones were then verified after amplification of the mutated region with primers PvdD(A1)_SeqF2 and PvdD(A1)_SeqR2 (Additional file 1: Table S2) followed by Sanger sequencing. For the deletion of both genes *fpvA* and *fpvB* in PAO1Δ*pvdF*Δ*pchA*, we proceeded with the method described in a previous study [66]. All strains and plasmids used in the study are listed in table S3.

### Growth rate and PVD production determination

PaM1 and all constructed mutants were cultivated in casamino acid medium (CAA) containing casaminoacids (5 g/L), K_2_HPO_4_ (6.4 mM) and MgSO_4_ (1 mM), or in minimal medium succinate (MMS) containing K_2_HPO_4_ (32.5 mM), KH_2_PO_4_ (22 mM), MgSO_4_ (811.4 µM), (NH_4_)_2_SO_4_ (7.57 mM), Succinic acid (33.87 mM) and NaOH (77.5 mM). The overnight cultures were conducted at 30 °C and orbital agitation at 220 rpm. The next day, cultures were centrifuged and the cell pellets were washed twice with fresh medium. A second overnight culture at 30 °C was started by resuspending the pellet in 10 mL of fresh medium supplemented with 0,5% arabinose to induce PVD production. The overnight culture was centrifuged and washed once with fresh medium. To determine growth rate and PVD production, cultures were inoculated either in CAA, or in MMS medium supplemented or not with 1.5 mM 4-azido-l-homoalanine (JenaBioscience, Jena, Germany), in a 96-well sterile plate (for growth comparison) or a 50 mL falcon tube (for PVD production determination), to an initial cell rate of 10^7^ CFU/mL. All cultures were grown at 30 °C and supplemented with arabinose 0.5% to induce PVD production. Absorbance at 600 nm (A600) representing bacterial turbidity was monitored every ½ h for 48 h in a TECAN Plate reader using Magellan software. PVD production was monitored by measuring the absorbance at 400 nm (A400) using the extinction coefficients ε = 19,000 (mol L)^−1^ cm^−1^ [26, 67]. After 48 h, the cultures were centrifuged at 8500 RPM for 5 min and the supernatant was filter-sterilized twice using 0.22 µm Millex-GP filters (Merck, Darmstadt, Germany). For mass spectrometry analysis, PVD was purified from the supernatants by using the Amberlite XAD4 polymeric resin (Merck, Darmstadt, Germany) and eluted with H_2_O/Ethanol (50/50).

### Characterization of PVD by mass spectrometry

MALDI Mass Spectrometry analyses were performed on an Autoflex Speed II (Bruker) in a reflectron acquisition mode. α-cyano-4-hydroxycinnamic acid at 10 mg/mL in H_2_O/ACN/TFA (50/50/0.3) was chosen as the matrix. Tandem Mass Spectrometry analyses were performed by ESI–MS on a MicrOTOF Q-II (Bruker) instrument. Calibration was carried out in the positive ion mode using the calibration solution provided by the manufacturer (Tune Mix Low, Bruker) over a mass range of 322–1822 m*/z*. Prior to ESI–MS experiments, PVD were desalted and diluted at 5microM in H_2_O/ACN/HCOOH (50/50/0.1). Doubly-charged ions were chosen as precursors and the collision energy was adjusted to maximize the coverage in fragment ions from 30 to 50 eV with 5 eV increment. Characteristic b- and y-fragments confirmed the PVD structure (Table S1). Y-fragments containing the C-terminal peptide ring were detected at low collision energy only (30–35 eV).

### Titration experiments

In order to estimate the proportion of both species in the wild-type strain, a titration experiment was carried out by MALDI-MS, testing the MS-response factor of these two forms of PVD (Additional file 1: Fig. S5). The intensity ratios between the *m/z* 1320.7 and *m/z* 1334.7 ions were calculated and a good linearity was obtained (R^2^ = 0.9905). PVD produced by PaM1 and Phe191Val obtained from calibrated CFU/mL at 5 × 10^8^ after 48 h of culture were used to determine the response factor of PVD-Ser compared to PVD-Thr and the proportion of PVD-Ser and PVD-Thr produced by the strains PaM1 and its derived mutant Phe191Val. The PVD of each strains were then compared by MALDI-MS directly in the bacterial medium without extra purification step. For the calculation of PVD-Ser to PVD-Thr ratios, the peak intensities of the entire isotopic profile of the main signal at *m/z* 1320 and 1334 were summed with the peak intensities of their corresponding retro-Diels Alder reaction at *m/z* 1017 and 1031 respectively.

### Copper-free cycloaddition of DBCO-oxazolidinone with PVD-azHA and conjugate purification

DBCO-oxazolidinone (DBCO-ox) was synthesized as described in Additional files 1 Fig. S11 to S15 and 2). Since PVD was shown to chelate copper ions [68], SPAAC was preferred in a first approach [21]. One liter of MMS medium was inoculated with the mutant strain His299Ala at an initial cell rate of 10^7^ CFU/mL in the presence of 1.5 mM of 4-azHA and cultivated for 48 h with orbital agitation (220 RPM) at 30 °C. The PVD mixture containing the PVD-azHA was purified as described previously and lyophilized, and the total amount of PVD produced was weighed. With the approximation that PVD-azHA represented at top 10% of total PVD, we adjusted its concentration to 2.2 mM in purified water. We added 1.5 equivalents of the DBCO-ox previously dissolved in DMSO (at 3.3 mM) to a final DMSO concentration of 5%. The mixture was incubated 48 h at room temperature (18–26 °C). The product of SPAAC reaction was lyophilized to remove the reaction solvent and dissolved in 1 mL H_2_O/ACN (50/50). The PVD-oxazolidinone conjugate was separated from the non-conjugated PVD by using a Size-exclusion chromatography column of 38 × 2.5 cm, with a polyacrylamide beads Bio-Gel P2 Extra Fine matrix (BioRad, Hercules, USA) in H_2_O/ACN (50/50) as the elution solvent. Fractions containing separated PVD-oxazolidinone were pooled and lyophilized. The total amount of conjugate recovered was 4.9 mg. It was completely resuspended in DMSO at a stock concentration of 1 mM for downstream Chrome-azurol S, growth and MIC assays.

### Chrome-Azurol S assay

The capacity of the conjugate PVD-oxazolidinone (**7**) to chelate iron was assessed by using a colorimetric Chrome-Azurol S (CAS) assay developed by Schwyn and Neilands [69]. Briefly, a CAS solution was prepared (HDTMA 600 µM, CAS 150 µM, anhydrous piperazine 500 mM in HCl 2 M) and complexed (CAS) or not (Apo-CAS) with iron from a FeCl_3_ 15 µM solution in HCl 10 mM. 4 mM of sulfosalicylic acid was added to reduce the reaction incubation time. In a 96-well microplate, 100 µL of the CAS reagent was added, using Apo-CAS as the positive control, and mixed with 100 µL of 1, 10 or 100 mM of PVD-Thr (dissolved in water) or PVD-ox (dissolved in DMSO/H_2_0 50%). To allow the siderophores to chelate iron and remove it from CAS, causing a coloration shift of the medium, we incubated the plate at 25 °C for 30 min. We monitored the coloration change by measuring OD at 630 nm.

### Minimum inhibitory concentration (MIC) determination

For MIC determination, the microdilution assay was used in a 96-well U-bottom microplate (Greiner Bio-one). Strains PAO1 and PAO1Δ*pvdF*Δ*pchA* were cultivated overnight in LB at 30 °C and the next morning, the culture was washed twice using fresh CAA medium. A new culture was started at OD 600 = 0.05 in CAA to induce expression of the pyoverdine transporters. When OD 600 = 0.5 was reached, the cultures were diluted to 10^6^ CFU/mL. In the microplate, microdilutions of Linezolide, DBCO-ox or PVD-oxazolidinone were added at final concentrations ranging from 512 to 0.5 µM in ½ successive dilutions. The wells were inoculated to a final turbidity of 5.10^5^ CFU/mL and the plate was incubated at 37 °C for 18 h before reading the OD 600 nm.

### Growth assay in iron-deficient conditions

Growth assays to determine the capacity of *P. aeruginosa* PAO1 to use the conjugate PVD-oxazolidinone as a siderophore were performed in a U-bottom 96-wells microplates (Greiner Bio-One, Kremsmünster, Austria). The strain PAO1Δ*pvdF*Δ*pchA*, unable to produce its own siderophores PVD and pyochelin [66], as well as the strain PAO1Δ*pvdF*Δ*pchA*Δ*fpvA*Δ*fpvB* lacking both PVD transporters (this study) were grown as described above: a first overnight culture at 30 °C in 5 mL LB broth, followed by washing of the bacteria and a second overnight culture in 5 mL CAA medium at 30 °C. Bacteria were then washed, resuspended in CAA medium at an optical density of 0.01 at 600 nm, and distributed in the wells of the plate in the presence or absence of 20 μM PVD or PVD-ox. The plate was incubated at 30 °C, with shaking, in an Infinite M200 microplate reader (Tecan, Männedorf, Switzerland) and bacterial growth monitored (DO600). The presented data are the mean of three replicates for each measurement.

## Computational section

### A-domain amino-acid sequence alignments

1546 A-domain sequences and their respective substrate were exported from the annotated NRPS substrate predictor database [70]. The sequences were sorted in two categories: Threonine-specific (T) A-domains (39 sequences) and non-threonine specific (NT) A-domains (1507 sequences). The sequences were aligned twice using clustalW and MUSCLE with the help of the SnapGene software (SnapGene v7.0.2). Sequences harboring another amino acid than an l-Asparate at position 190 were removed from the list for homogeneity, leaving 38 T and 1196 NT remaining A-domain amino acid sequences. All possible residues from the 20 proteinogenic amino acids at the 8 residues of the “specificity code” were compared and their frequency was expressed in %. A threshold to consider a residue as a “key residue” was set on > 90% conservation in T A-domains and < 5% represented in NT A-domains.

### PvdD(A1) 3D model construction

At the time of the study, AlphaFold 2 was not available, therefore, the classical method of 3D model construction by homology was carried out. The genbank-ID: OPE27038 sequence from residue Phe508 to Ser1013 was used as a BLASTP query (with default values) to search the Protein Data Bank (PDB) [71] for homologous and resolved experimental structures that could serve as templates for 3D model construction. Structural chains that showed a score greater than 250 and an identity percent greater than 39 were selected, *i.e.* the 56 top hits, while the expect values ranged from 10^–140^ to 2 × 10^–75^. PDB entries exhibiting a resolution greater than 2.5 Å were discarded and 35 structures remained. The Protein Structural Statistic (PSS) program was then used to superimpose the selected 3D structures [72, 73]. Seventeen structures presented a folded region that shared homology with the query sequence C-terminus, *i.e.* from residue Leu940 to Ser1013 while 18 did not. The 17 sequences were aligned with the query using clustalω [74] and a phylogenetic tree was constructed with MegaX [75] and the Neighbour-Joining algorithm [76] (Substitution model JTT and 500 bootstrap replicates). In the phylogenetic tree (Additional file 1: Fig. S10), two groups of sequences were close to the *P. aeruginosa* PvdDA1 query sequence, *i.e.* 5N9W/X 10.2210/pdb5N9W/pdb, 10.2210/pdb5N9X/pdb and 3VNR/S/Q, 10.2210/pdb3VNS/pdb10.2210/pdb3VNQ/pdb, 10.2210/pdb3VNR/pdb. Finally, 5N9X structure was selected as a template to build the model, *i.e.* the adenylation domain of *Streptomyces SP.OH-5093* involved in the biosynthesis of 4-chlorothreonine, since it was bound to threonine, which is also the natural substrate of *P. aeruginosa* PvdDA1. Chain A of PDB-ID:5N9X from residue Asp22 to Arg529 was used as a template to build models with I-Tasser [77] and SwissModel [78] on-line servers and Modeller [79]. Generated models were then submitted to SAVES 6.0 [80] to check their quality and Ramachandran plots were produced. For the SwissModel 3D molecular construct, 92%, 6.8% and 0.9% residues showed dihedral angles in most favored regions, additional allowed regions and generously allowed regions of the plot, respectively. Models generated by I-Tasser or Modeller exhibited lesser percentages. Therefore, the SwissModel was selected for further refinement. Protonation states of titratable residues were determined at pH 7.4 with the PROPKA program implemented in the PDB2PQR server [81]. Hydrogen atom placement on the protein was performed using the HBUILD [82] facility in the CHARMM program [83]. The model energy was then minimized with 500 steps of steepest descent algorithm using CHARMM program version c37b1 with non-bonded interactions truncated at 14 Å distance using switch and shift functions for van der Waals and electrostatic forces, respectively. In our study, the first residue of the A1-domain corresponds to the residue Arg507 of the full PvdD. Point mutations were introduced in silico to the homology model structure using the SCWRL4 program [84]. Ligands l-Thr and 4-azHA were docked by comparative docking with respect to the structure 5N9X. An initial set of force field parameters for 4-azHA was obtained from the Paramchem webserver [85] and the CGenFF [86] and refined both manually and by quantum mechanical calculations using the Gaussian program [87]. A model of the full-length protein has been made available on the AlphaFold database [88]. First, both homology-based and AlphaFold PvdDA2 domains were superposed. A root mean square deviation (RMSD) of 1.47 angströms was calculated on 506 residues and 3037 atoms (Fig. S17a). Second, a ten angström sphere was drawn around the Thr substrate and RMSD was determined for the residues inside the sphere (Fig. S17b); 57 residues and 327 atoms were involved and the superposition produced a 0.44 angströms RMSD. Finally, a five angström sphere was drawn around the Thr substrate which included 15 residues and 122 atoms. The RMSD was determined for these residues (Annex Fig. 1c), i.e. 0.52 angströms. In conclusion, both models superpose very well and particularly on the substrate binding pocket.

### Molecular dynamics simulations

#### MD simulations and free-energy decomposition analysis

##### MD simulations

The homology models of the WT and the mutant PvdD(A1) described above were used as starting points for the MD simulations. The titration states of all histidines in the complexes were determined at pH 7.4 with the PROPKA program implemented in the PDB2PQR server [81]. These protonation states were taken into consideration when adding hydrogen atoms to the crystal structure using the HBUILD module in the CHARMM program [83]. The CHARMM36 all-atom parameter set [89] was used. Each complex was subjected to the following protocol. Except for ligand, the entire complex was initially held fixed and an energy minimization using the steepest descent method was applied for 500 steps. This allowed the ligand to adjust to the binding pocket. The constraints were removed and the full complex was minimized for another 500 steps by steepest descent. The complexes were solvated in a 120 Å cubic box of TIP3P water, sodium ions were added to neutralize the overall charge and additional NaCl pairs were added to give an ionic strength of about 0.15 M. Molecular dynamics simulations were done using the NAMD program [90]. The protein complex was then fixed and the system was energy minimized by 1000 steps by the conjugate gradient method and the heated from 0 to 600 K, incrementing the temperature by 0.5 K each 10 fs. A second energy minimization of 250 steps and heating cycle from 0 to 300 K by steps of 0.25 K every 10 fs were performed to better position water molecules around the complex. The constraints on the complex were then released and the entire system was energy minimized for 2000 steps followed by a heating simulation from 0 to 300 K using the same settings as above. The system was equilibrated for 15 ps at 300 K before the beginning of the production phase. The simulations were carried out under NVE conditions with periodic boundary conditions and Ewald summation. The production phases for all complexes were carried out for 100 ns each. For each system, 6 replica simulations were run.

##### Free-energy decomposition analysis

Estimates of the individual amino acid contributions to the binding free energy related to ligand binding were obtained from structures representing the conformational space sampled during the MD simulations. 100 structure were extracted along the MD trajectory and the free-energy analysis was performed using the MM/PBSA free energy decomposition scheme using in-house programs [91].

### Supplementary Information

Additional file 1.

Additional file 2.

## Data Availability

The datasets used and/or analysed during the current study are available from the corresponding author on reasonable request.
