# Supplementary material for: Biosynthesis of a clickable pyoverdine via in vivo enzyme engineering of an adenylation domain
Source: Microb Cell Fact. 2024 Jul 24;23:207. doi: 10.1186/s12934-024-02472-4 (PMC11267755; doi:10.1186/s12934-024-02472-4)
Supplement: Supplementary file 1 — Additional file 1. [file 12934_2024_2472_MOESM1_ESM.docx]

**Additional file 1**

**Biosynthesis of a clickable pyoverdine via in vivo enzyme engineering of an adenylation domain**

Hélène PUJA, Laurent BIANCHETTI, Johan REVOL-TISSOT, Nicolas SIMON, Anastasiia SHATALOVA, Julian NOMME, Sarah FRITSCH, Roland H. STOTE, Gaëtan L.A. MISLIN, Noëlle POTIER, Annick DEJAEGERE, Coraline RIGOUIN.

# **Supplementary tables and figures**

| Fragment | PVD-Thr  Parent ion : 667.8 | PVD-Ser  Parent ion : 660.8 | PVD-Val  Parent ion : 666.8 | PVD-Leu/Ile  Parent ion : 673.8 |
| --- | --- | --- | --- | --- |
| a1 | 417.14 | 417.141 | 417.137 | 417.137 |
| a1-H2O | 399.1305 | 399.132 | 399.126 | 399.129 |
| b1 | 445.138 | 445.142 | 445.125 | 445.136 |
| b1-H2O | 427.118 | 427.127 | - | 427.127 |
| b2 | - | 601.242 | - | - |
| b2-17 | 584.215 | 584.219 | 584.214 | 584.214 |
| b3 | 688.267 | 688.271 | 688.265 | 688.27 |
| b3-17 | 671.242 | 671.247 | 671.239 | 671.239 |
| b3-H2O | 670.226 | 670.26 | 670.252 | 670.252 |
| b4 | 846.335 | 846.339 | 846.323 | 846.339 |
| b4-17 | 829.31 | 829.313 | 829.317 | 829.311 |
| b4-H2O | 828.322 | 828.324 | 828.33 | 828.33 |
| b5 | 974.43 | 974.436 | 974.43 | 974.43 |
| b5-17 | 957.404 | 957.407 | 957.395 | 957.414 |
| y3 | 361.171 | 347.152 | - | - |
| y4 | 489.263 | 475.248 | 487.283 | 501.299 |
| y6 | 734.367 | 720.345 | 732.388 | 746.381 |
| y7 | 890.465 | 876.459 | 888.521 | 902.514 |

**Table S1.** Theoretical *m/z* values masses expected after fragmentation of the parent ions of PVD-Thr (*m/z* 667.8), PVD-Ser (*m/z* 660.8), PVD-Val (*m/z* 666.8) and PVD-Leu/Ile (*m/z* 673.8) by ESI MS-MS. Highlighted in red are shown the characteristic ions allowing to identify the mass shift of the respective species.

**Table S2**. Primers used in this study.

**Table S3.** Strains and plasmids used in this study.

**Figure S1.** Amino-acid frequency of specificity-conferring residues in A-domains. The frequency of each residue of PvdD at the 8 specificity-conferring positions was obtained by Clustalω sequence alignments. The distribution (in %) is shown at each of the positions in Threonine-specific A-domains (Black bars) compared to non-threonine A-domains (White bars). The threshold represented as dashed lines shows a frequency higher than 90% (higher bar) or lower than 5% (lower bar).

**Figure S2.** Growth curves of the strain PaM1 and mutants. The growth of the F191A, F191V, F191I, H299A, H299C and H299N mutants is compared to the strain PaM1 for 40 hours **(a)** in the iron-deficient CAA medium and (**b)** in MMS minimal medium supplemented (blue) or not (orange) in 4-azHA

**Figure S3.** ESI-MS/MS experiments of natural and modified PVD. The MS/MS spectra display the fragmentation pattern obtained after CID of (a) *m/z* 667.8 precursor ion corresponding to PVD-Thr, (b) *m/z* 673.8 precursor ion corresponding to PVD-Leu/Ile and (c) *m/z* 666.8 precursor ion corresponding to PVD-Val. Zoom in the *m/z* 350-510 and 680-980 range are shown here to better visualize the mass shift of characteristic ions y3, y4, y6 and y7 discriminating the different species (highlight in red).


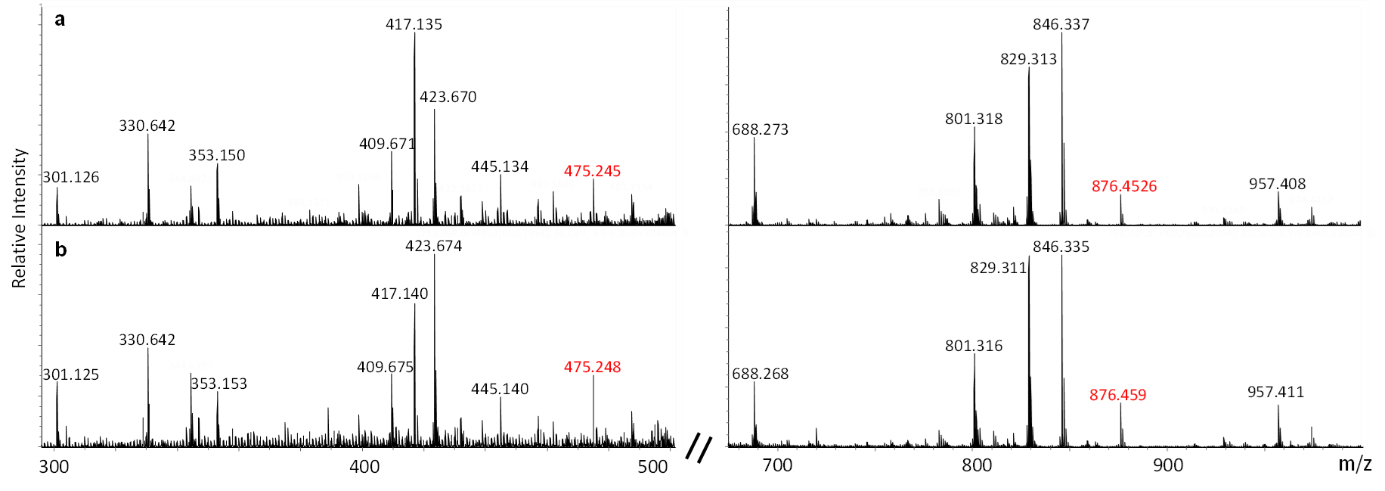


**Figure S4.** ESI-MS/MS of the *m/z* 660.8 ions corresponding to PVD-Ser, produced by (a) the strain PaM1 or (b) the mutant strain Phe191Val. In red are highlighted the characteristic ions y4 and y7 specific for the PVD-Ser species.

**Figure S5.** Titration experiment using calibrated bacterial density at 5x10^8^ after 48h of culture to determine the response factor of PVD-Ser compared to PVD-Thr (highlighted in red) and the proportion of PVD-Ser and PVD-Thr produced by the strains PaM1 and its derived mutant Phe191Val. The two upper spectra were obtained directly from the culture media without any purification to preserve the production proportions while the lower spectrum was obtained from an equivalent mixture of both purified PVD species. For the calculation of PVD-Ser to PVD-Thr ratios, the peak intensities of the entire isotopic profile of the main signal at *m/z* 1320 and 1334 were summed with the peak intensities of their corresponding retro-Diels Alder reaction at *m/z* 1017 and 1031 respectively.

**Figure S6.** MALDI-MS of the PVD produced by the mutants Phe191Ile (a), Phe191Ale (b), His299Asn (c) and His299Cys (d).


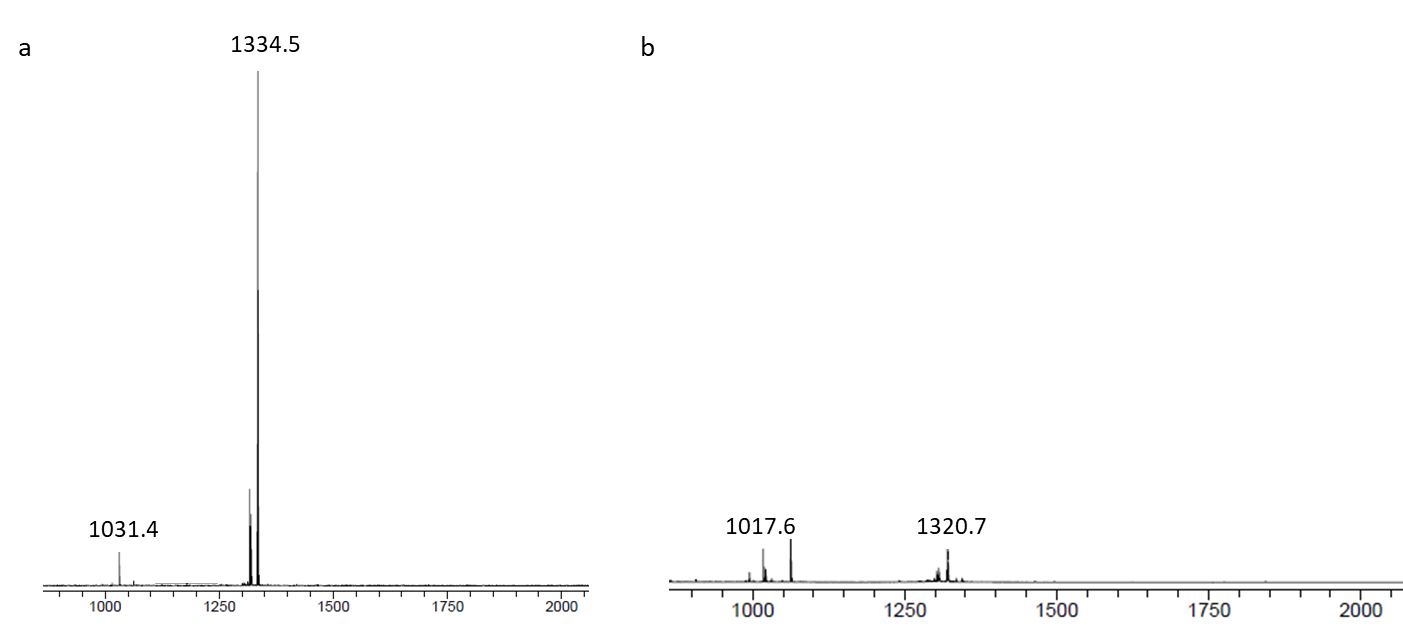


**Figure S7.** MALDI spectra of the PVD produced by the strain PaM1 (a) and the mutant Phe191Val (b) grown in MMS medium

**Figure S8.** 3-D modeling of the impact of His299Ala mutation on 4-azHA recognition by the PvdD(A1) domain. (a.) Binding site of the wild-type PvdD(A1) complexed with 4-azHA. The cavity between the substrate 4-azHA and the interacting residues forming the binding pocket is represented as a purple mesh surface. (b.) Binding site of His299Ala PvdD(A1) complexed with azido-alanine. (c.) Free binding energy calculated by MM/PBSA, total or by amino-acid. The residue His299 in PaM1, corresponding to an Ala in the His299Ala mutant, is highlighted in yellow, and the catalytic residues are highlighted in gray. N.I.: Not involved, meaning that there is no significant interaction of the residue with the substrate. (d.) RMSF fluctuations for the wild-type PvdD(A1) docked with 4-azHA. (e.) RMSF fluctuations for the mutant His299Ala docked with 4-azHA. The results are represented as a heat map projected onto the backbone structure, the blue residues being the less flexible and the red being more flexible

**Figure S9.** Mass spectrometry detection of the PVD-oxazolidinone conjugate after click chemistry. (a) MALDI spectrum of purified pyoverdines before click-chemistry. (b) Zoom of the spectrum (a) showing the different pyoverdines in the mixture, including the PVD-azHA highlighted in red. (c) MALDI spectrum of the purified pyoverdines after click-chemistry. The PVD-oxazolidinone conjugate is highlighted in red. (d) zoom of the spectrum (c) showing the disappearance of PVD-azHA.


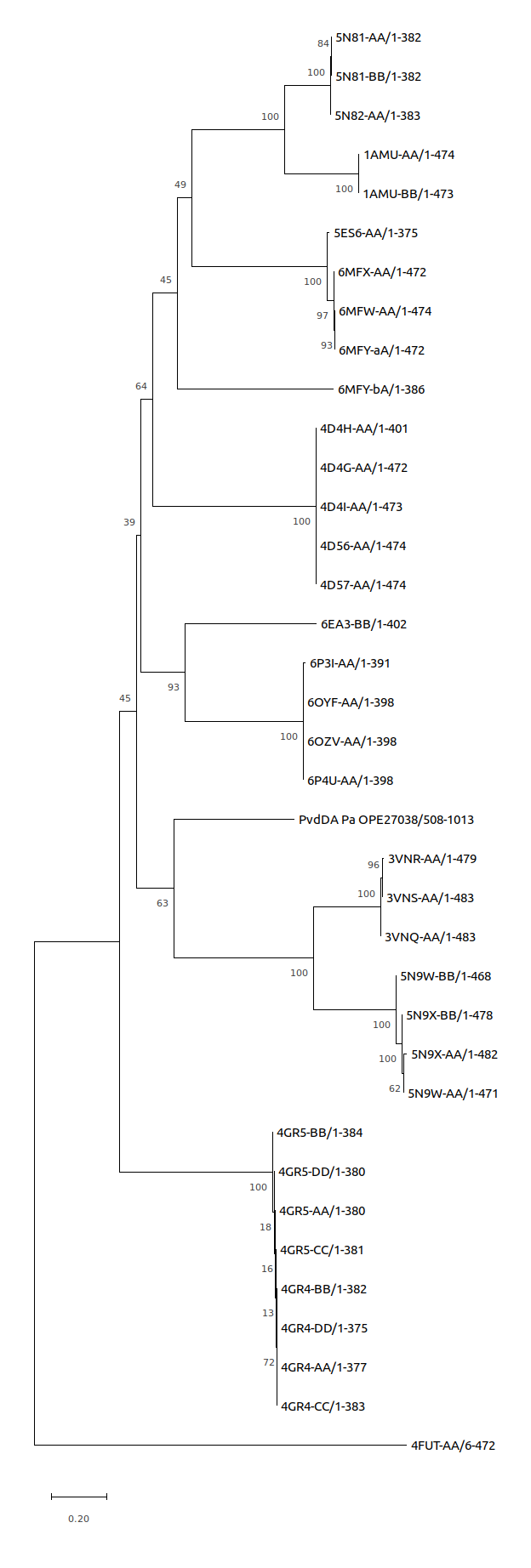


**Figure S10:** The phylogenetic tree developed for the homology modeling


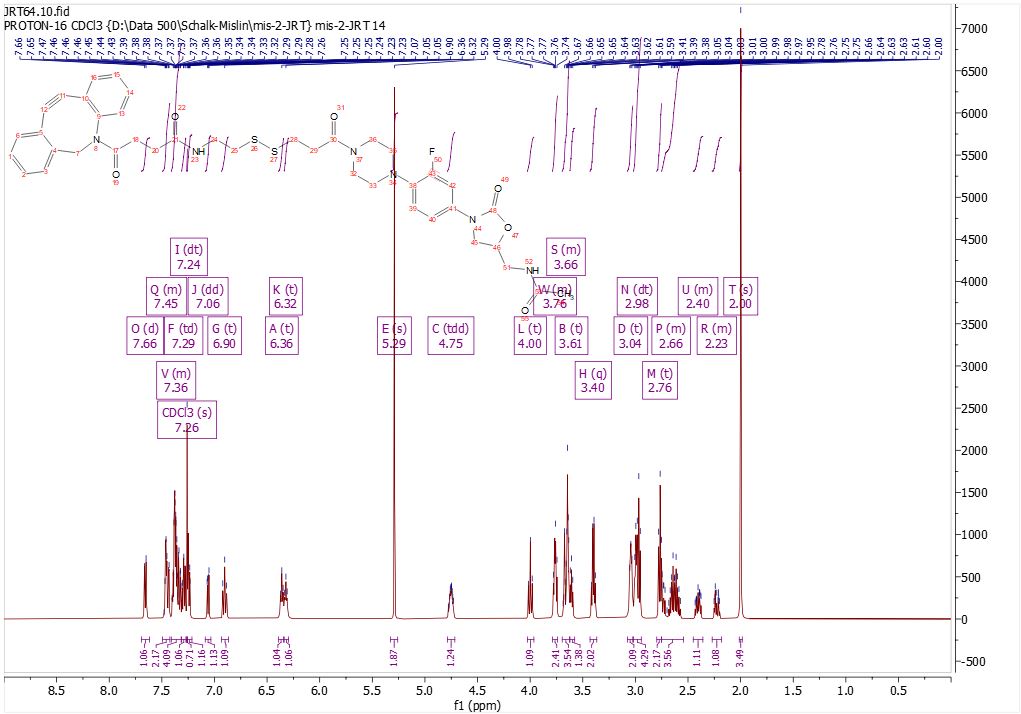


**Figure S11:** ^1^H-NMR spectrum of compound DBCO-ox (CDCl_3_, 500 MHz)


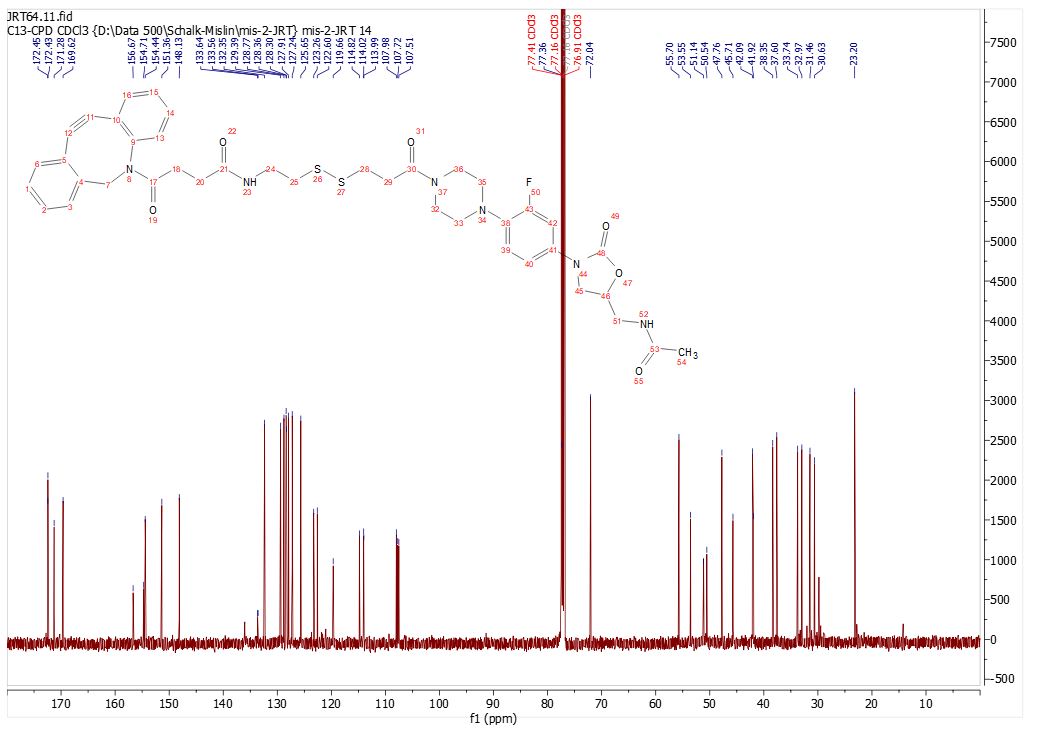


**Figure S12.** ^13C^-NMR spectrum of DBCO-ox (CDCl_3_, 126 MHz)


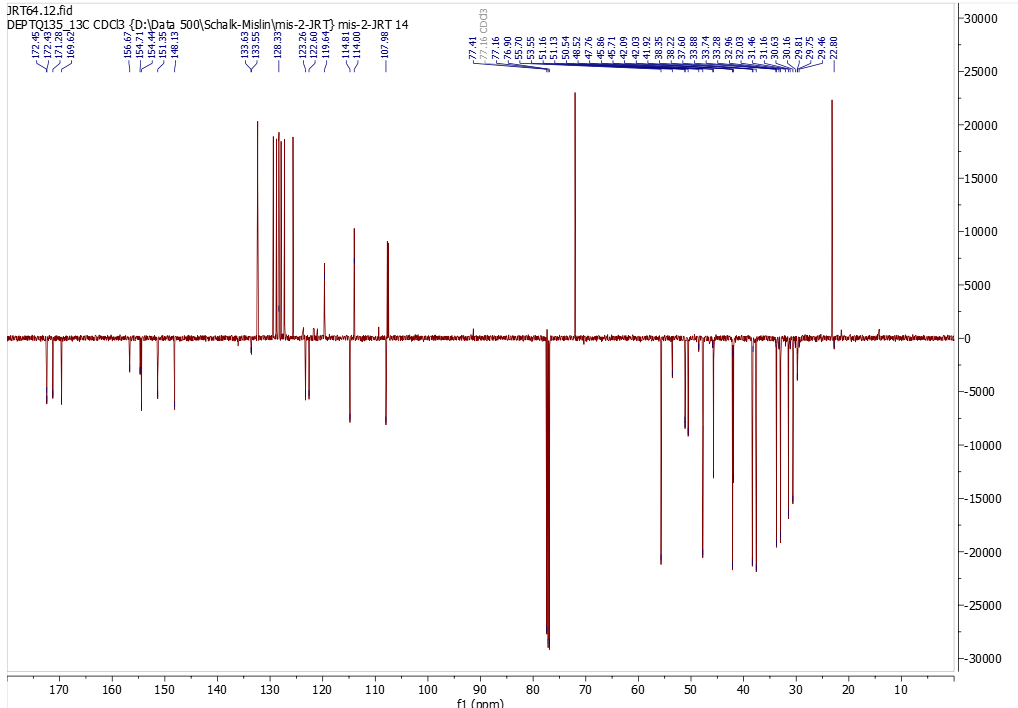


**Figure S13.** ^13^C-NMR (DEPTQ) spectrum of DBCO-ox (CDCl_3_, 126 MHz)


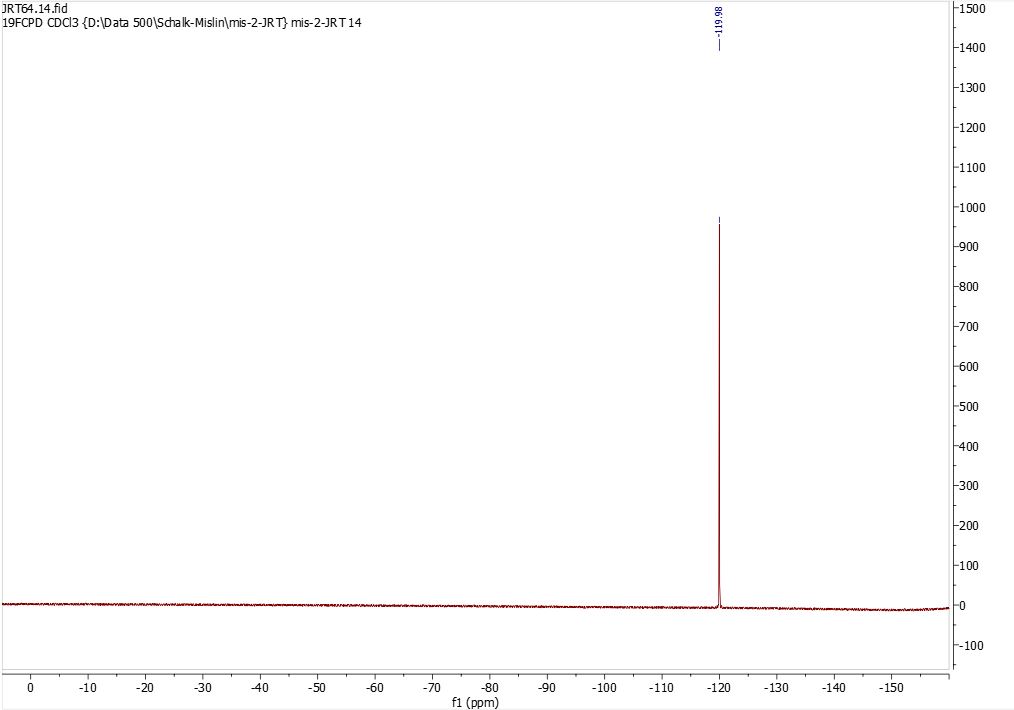


**Figure S14.** ^19^F-NMR spectrum of DBCO-ox (CDCl_3_, 471 MHz)

**
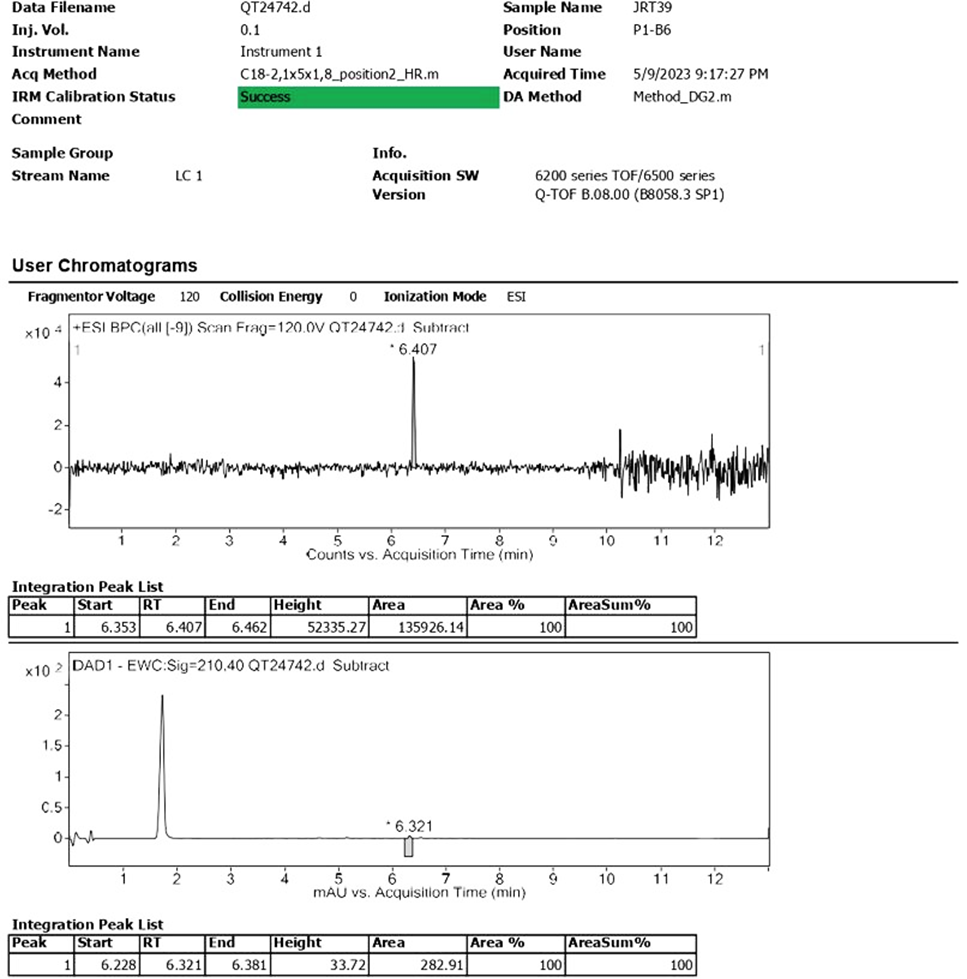
**

**
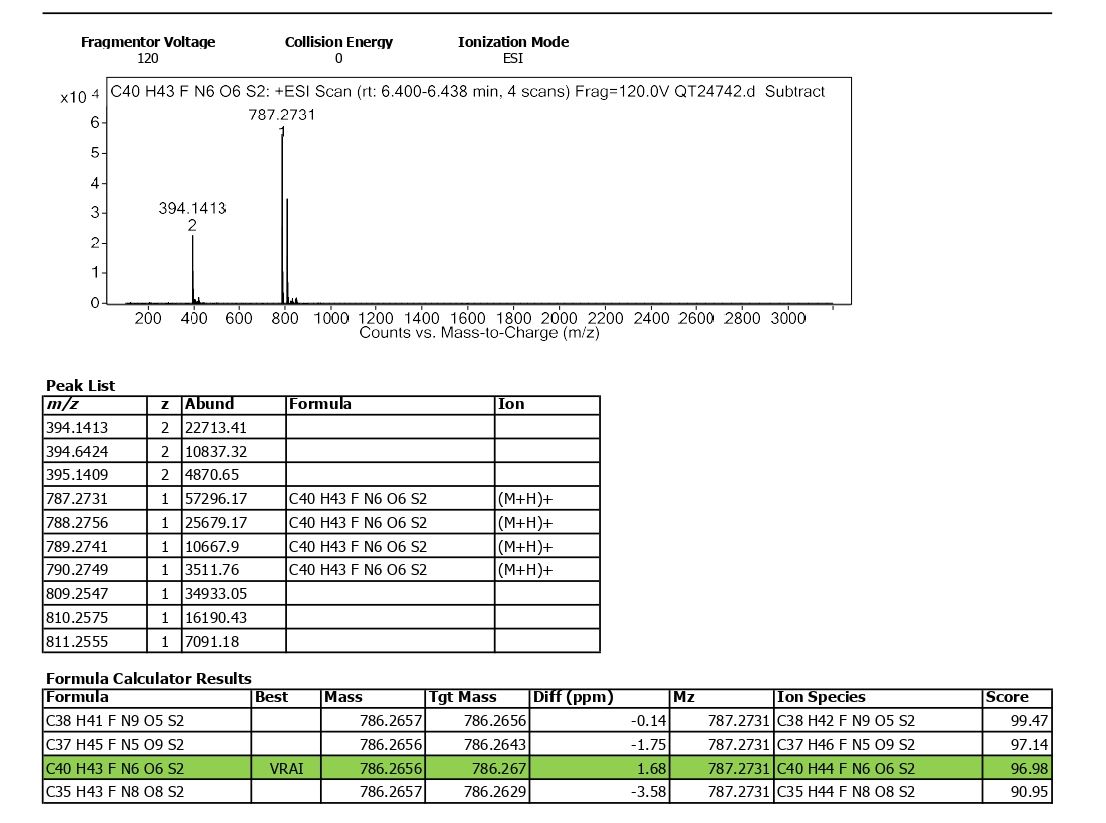
**

**Figure S15**. LC-HRMS Spectrum of DBCO-ox

**Figure S16:** Synthesis of DBCO-ox

Figure S17: Superposition of the PvdDA2 models generated by homology (blue) and AlphaFold 2 (red). The threonine substrate is shown in sticks. (a) Whole PvdDA2 domain, polypeptide backbones are shown as ribbon. (b) Backbone representation of residues within a ten angström radius sphere centered on the Thr substrate. (c) Residues within a five angström radius sphere around the Thr substrate, side chains are represented with lines.
