# Supplementary material for: Biosynthesis of a clickable pyoverdine via in vivo enzyme engineering of an adenylation domain
Source: Microb Cell Fact. 2024 Jul 24;23:207. doi: 10.1186/s12934-024-02472-4 (PMC11267755; doi:10.1186/s12934-024-02472-4)
Supplement: Supplementary file 2 — Additional file 2. [file 12934_2024_2472_MOESM2_ESM.docx]

# **Additional file 2**

**Biosynthesis of a clickable pyoverdine via in vivo enzyme engineering of an adenylation domain**

Hélène PUJA, Laurent BIANCHETTI, Johan REVOL-TISSOT, Nicolas SIMON, Anastasiia SHATALOVA, Julian NOMME, Sarah FRITSCH, Roland H. STOTE, Gaëtan L.A. MISLIN, Noëlle POTIER, Annick DEJAEGERE, Coraline RIGOUIN.

# **Supplementary methods**

**Synthesis of DBCO-oxazolidinone.**

Synthesis of DBCO-ox was synthesized according to Supplementary Fig. S15 using oxazolidinone **1** and DBCO derivative **2.** Compound **1** was synthesized according a previously described protocol [46,47]. DBCO derivative **2** was purchased to *tebubio Fance* (Le Perray en Yvelines, France). Other more common chemicals were purchased from *TCI Chemicals*, *Merck-Sigma-Aldrich* or *Acros-Thermo Fischer*. Solvents were purchased from *Carlo Erba*. All reactions were carried out under argon (technical quality, Air products). Solvents used were of analytical grade purity (>99.9%). When necessary, solvents and bases were purchased extra-dry. All other chemicals were used as received, unless otherwise stated.

All reactions were monitored by thin-layer chromatography (TLC) using *Merck* precoated silica gel 60F^254^ (0.25 mm). TLC are visualized using UV (254 nm/365 nm, *Vilber Lourmat*, VL-4LC, Eberhardzell, Germany) and/or using classical revelation mixtures (sulfuric vaniline, potassium permanganate, ninhydrin reagent). Before chromatographic purification reaction mixtures were adsorbed on silica gel (60-200 µm, VWR Chemicals). Chromatographic purifications were performed on a Selekt Enkel (*Biotage* Uppsala, Sweden) purification device using PuriFlash® pre-packed silica gel columns (*Interchim*, Montluçon, France).

Nuclear Magnetic Resonance analysis of DBCO-ox and spectroscopic analysis: NMR spectra were recorded on Bruker Avance 400 (1H: 400 MHz, 13C: 100 MHz, 19F: 376 MHz) or Avance 500 (1H: 500 MHz, 13C: 125 MHz, 19F: 470 MHz), using the residual non-deuterated solvent as reference. The chemical shifts (δ) and coupling constants (J) are expressed in ppm and hertz respectively. Multiplicity is indicated as follow: s for singlet, d for doublet, t for triplet, q for quadruplet, quint for quintuplet and m for a multiplet. Mass spectra were recorded in the Service Commun d’Analyses (SCA) de la Faculté de Pharmacie de l’Université de Strasbourg LC/BRMS conditions : Analytical RP-HPLC-MS was performed using a LC 1200 Agilent with quadrupole-time-of-flight (QTOF) (Agilent Accurate Mass QToF 6520) with a Zorbax Agilent C18-column (C18, 50 mm x 2.1 mm; 1.8 µm) using the following parameters: The solvent system: A (acetonitrile + 0.05% formic acid) and B (H2O + 0.05% formic acid); Gradient (Tmin,(%B)): T0 (98%), T4(0%), T8(0%), T8.1(98%). Flow rate of 0.5 mL/min; Column temperature: 40°C; DAD scan from 190 nm to 700 nm. Ionization mode: ESI+. LC/HRMS conditions: Analytical RP-HPLC-HRMS was performed using a LC 1200 Agilent with quadrupole-time-of-flight (QToF) (Agilent Accurate Mass QToF 6520) with a Zorbax Agilent C18-column (C18, 50 mm x 2.1 mm; 1.8 µm) using the following parameters: The solvent system: A (acetonitrile + 0.05% formic acid) and B (H2O + 0.05% formic acid); Gradient: (Tmin, (%B)): T0 (98%), T8(0%), T12.5(98%), T12.6(98%), T13(98%). Flow rate of 0.5 mL/min; Column temperature: 40°C; DAD scan from 190 nm to 700 nm. Ionization mode: ESI+. For HRMS, the calculated and the found mass correspond to M. Original ions repartition could be found in ions table and simulation given Additional file 1, Fig. S10, S11, S12, S13 and S14.

**4-{2-azatricyclo[10.4.0.0^4,9^]hexadeca-1(12),4(9),5,7,13,15-hexaen-10-yn-2-yl}-N-(2-{[3-(4-{4-[5-(acetamidomethyl)-2-oxo-1,3-oxazolidin-3-yl]-2-fluorophenyl}piperazin-1-yl)_3-oxopropyl]disulfanyl}ethyl)-4-oxobutanamide (DBCO-ox)**: In a first step, the Boc-oxazolidinone derivative **1** (82 mg, 0.19 mmol) was deprotected using a mixture CH_2_Cl_2_/TFA 80/20 (3.5 mL). The solution was stirred at 27°C till the total consumption of the starting material (about 5h), checked with TLC. Solvent and TFA were then evaporated under reduced pressure. The resulting oxazolidinone compound **3** was not purified and used as it is for the next step. In parallel, DBCO derivative **2** (90 mg, 0.19 mmol) and pentafluorophenol (71 mg, 0.38 mmol) were solubilized in CH_2_Cl_2_ (14 mL). EDCI hydrochloride (74 mg, 0.38 mmol) was added and the mixture was stirred at 27°C till the total consumption of the starting material (about 14h, overnight), checked by TLC. Volatile compounds were then removed from the mixture under reduced pressure to obtain DBCO-ester derivative **4** used as it is for the next step. In the last step, crude deprotected oxazolidinone compound **3** and crude DBCO-activated ester **l** were solubilized in CH_2_Cl_2_ (20 mL). DIPEA (393 µL, 0.29 mg, 2.27 mmol) and DMAP (5.1 mg, 0.042 mmol) were then successively added to the solution. Reaction mixture was stirred to reflux (38°C), till the total consumption of the starting material (about 24h), checked by TLC. Solvents were then removed from the mixture under reduced pressure. The crude was diluted in CH_2_Cl_2_ (100 mL) and successively washed with a 10% aqueous solution of citric acid (100 mL) and brine (100 mL). The organic layer was dried over Na_2_SO_4_, filtered, and solvent was evaporated under reduced pressure. The resulting yellow oil was then adsorbed on silica gel and purified by flash chromatography (12 g pre-packed silica gel column. Elution: gradient of A/B: 100/0 -> 0/100, using CH_2_Cl_2_ (A) and EtOH/CH_2_Cl_2_ 20% (B)). The expected DBCO-ox building block (130 mg, 0.17 mmol, yield: 87% over three combined synthetic steps) was isolated as a slightly-beige powder.

**^1^H NMR** (500 MHz, CDCl_3_) δ 7.66 (d, *J* = 7.5 Hz, 1H), 7.49 – 7.42 (m, 2H), 7.41 – 7.32 (m, 4H), 7.29 (td, *J* = 7.0, 1.1 Hz, 1H), 7.24 (dt, *J* = 7.3, 1.4 Hz, 1H), 7.06 (dd, *J* = 7.4, 1.4 Hz, 1H), 6.90 (t, *J* = 9.1 Hz, 1H), 6.36 (t, *J* = 6.2 Hz, 1H), 6.32 (t, *J* = 5.8 Hz, 1H), 5.29 (s, 2H), 4.75 (tdd, *J* = 8.8, 6.0, 3.2 Hz, 1H), 4.00 (t, *J* = 8.9 Hz, 1H), 3.79 – 3.74 (m, 2H), 3.70 – 3.63 (m, 4H), 3.61 (t, *J* = 6.0 Hz, 1H), 3.40 (q, *J* = 6.3 Hz, 2H), 3.04 (t, *J* = 5.0 Hz, 2H), 2.98 (dt, *J* = 14.2, 5.9 Hz, 4H), 2.76 (t, *J* = 6.9 Hz, 2H), 2.75 – 2.54 (m, 4H), 2.45 – 2.36 (m, 1H), 2.27 – 2.18 (m, 1H), 2.00 (s, 3H). **^13^C NMR** (126 MHz, CDCl_3_) δ 172.45, 172.43, 171.28, 169.62, 156.67, 154.71, 154.44, 151.36, 148.13, 136.02, 133.64, 133.56, 132.35, 129.39, 128.77, 128.36, 128.30, 127.91, 127.24, 125.65, 123.26, 122.60, 119.66, 114.82, 114.02, 107.98, 107.72, 107.51, 72.04, 55.70, 51.14, 50.54, 47.76, 45.71, 42.09, 41.92, 38.35, 37.60, 33.74, 32.97, 31.46, 30.63, 23.20. **^19^F NMR** (471 MHz, CDCl_3_) δ -119.98. **HRMS**: calculated for C_40_H_43_FN_6_O_6_S_2_: 786.2670; found: 786.2656.
